# A Pyrene- and Phosphonate-Containing Fluorescent Probe as Guest Molecule in a Host Polymer Matrix

**DOI:** 10.3390/molecules18021897

**Published:** 2013-02-01

**Authors:** Elise Villemin, Benjamin Elias, Michel Devillers, Jacqueline Marchand-Brynaert

**Affiliations:** Institute of Condensed Matter and Nanosciences, Molecules, Solids and Reactivity (MOST), Université catholique de Louvain, Bâtiment Lavoisier, place Louis Pasteur 1, L4.01.02, 1348 Louvain-la-Neuve, Belgium

**Keywords:** host-guest systems, pyrene luminescence, phosphonate, polymers

## Abstract

New host-guest materials have been prepared by incorporation of a home-made organic probe displaying a pyrene motif and a phosphonate function into a regular amphiphilic copolymer. Using powder X-Ray diffraction, photoluminescence and FT-IR spectroscopy, we have been able to study the non-covalent interactions between the host matrix and the guest molecule in the solid state. Interestingly, we have shown that the matrix directs the guest spatial localization and alters its properties. Thanks to the comparison of pyrene *vs.* *N*-pyrenylmaleimide derivatives, the influence of the chemical nature of the guest molecules on the non-covalent interactions with the host have been studied. In addition, using polyethylene glycol as a reference host, we have been able to evidence a true matrix effect within our new insertion materials. The phosphonated guest molecule appears to be a novel probe targeting the hydrophilic domain of the host copolymer.

## 1. Introduction

Supramolecular chemistry has received considerable interest in the past decade [1]. Non covalent interactions are used in order to assemble designed building blocks. These assemblies have various applications such as microreactors [2,3], organic or anionic species sensors [4,5], and materials for optical and electroluminescent devices [6,7,8].

Among the variety of optical sensors, the pyrene motif is often used for monitoring the polarity and viscosity of environments [9,10,11]. The formation of excimer species from pyrene derivatives allows, in solution, the detection of metallic cations by formation of supramolecular assemblies [12,13,14], the study of hybridization of complex systems such as nucleic acids [15,16], the dynamic investigation of macromolecules such as dendrimers and auto-assembled polymers [17,18]. Pyrene incorpored polymer films were prepared with the aim of creating new electronic or optical devices. The intensity of photoluminescence of pyrene (or monosubstituted pyrene derivatives) incorpored in films made from polyethylene [19], polypropylene [20], polystyrene [21,22], polyvinylalcohol [23], poly(methyl-methacrylate) [24,25] and sodium polyacrylate [23] increases with the increase of pyrene concentration. The thickness of the film and the pyrene concentration are the principal parameters for the modification of photoluminescence profiles [19,21,22]. The study of non covalent interactions inside pyrene-incorpored copolymers featuring different domains of polarity is not described in the literature so far. Moreover, no example describing a pyrene derivative used as sensor for solid materials has been previously reported. Pyrene-based sensors are only used in solution for the determination of domains in complex biological systems, such as biological polymers [26,27] and in enzymes [28]. In these cases, the sensor is designed in order to form electrostatic interactions (presence of a cationic motif) and/or hydrophobic interactions (presence of fluorescent pyrene motif and/or alkyl chains) with the biological system. The cationic motif possess a double function: it brings hydrophilicity to the sensor which becomes water-soluble and allows the creation of electrostatic interactions with negatively-charged guests. A few examples in the literature show that water-solubility may be brought by the functionalization of the pyrene nucleus with charged polar functions; for instance, pyrenes substituted by small dendrimers with multiple carboxylate functions are used as dispersants of single-walled-nanotubes in water [29]. The neutral phosphonate ester function has not been described before as a pyrene substituent in order to increase the hydrophilicity; pyrene substituted with a phosphonic acid group was used anecdotally for the elaboration of 2D supramolecular structure [30].

**Figure 1:**
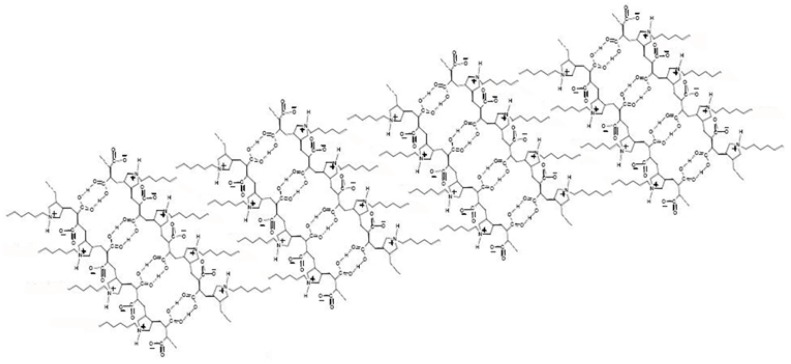
Schematic representation of the regular 3D-structure of the Copo C_6_-H polymer matrix.

Using an established Diels-Alder strategy [31,32,33], we have synthesized a novel phosphonated fluorescent probe (named CCNPyr) containing the pyrene motif. Our aim is to take advantage of the neutral phosphonate ester function to direct the probe into the polar regions of organic materials featuring an alternance of hydrophobic and hydrophilic micro-domains and to demonstrate the efficiency of this new phosphonated probe for the study of materials in the solid state. Accordingly, a complete study of the interactions between our probe (organic phosphonated guest molecule) and a zwitterionic host polymer, made by radical cyclo-copolymerisation of *N*-hexyl-*N*,*N*-diallylamine and maleic acid (named Copo C_6_-H, Figure 1) [34,35,36], has been performed in the solid state. This polyampholyte matrix was initially designed by one of us for the preparation of hybrid organic-inorganic materials by incorporation of inorganic salts in aqueous medium. For instance homogeneous hybrid blends in the solid state were obtained with charged coordination complexes as a result of electrostatic interactions principally and used as precursors for a wide series of multimetallic oxides based on elements of groups 5 and 6 [35,36].

A first series of “host-guest” materials Copo C_6_-H/CCNPyr was prepared and compared with materials similarly obtained using commercially available pyrene and pyrene derivative as guests. Our objective was to highlight a possible matrix effect of Copo C_6_-H due to its supramolecular organisation. The formation of microcavities and particularly the formation of less polar and more rigid microenvironments may enhance the photoluminescence of aromatic organic guest fluorophores, by shielding their excited species towards the quenching and nonradioactive transition that occur in a bulk solution [37,38]. Hence, a fluorescence increase could be expected for pyrene incorpored Copo C_6_-H materials.

**Figure 2:**
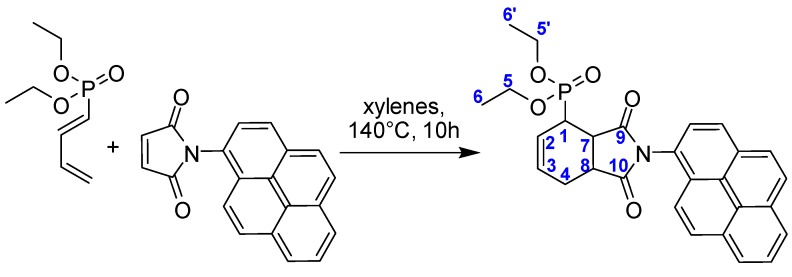
Synthesis of the organic guest molecule (CCNPyr) from 1-phosphono-1,3-butadiene and *N*-pyrenylmaleimide (NPyrMal).

A second series of “host-guest” materials has been prepared from a polar, but non-zwitterionic polymer, *i.e.* polyethyleneglycol (PEG-5000), and CCNPyr. PEG-5000 is a semi-crystalline polymer which presents amorphous domains, noted soft domains, and crystalline domains, noted rigid domains. The fluorescence properties of these materials PEG-5000/CCNPyr compared to the corresponding Copo C_6_-H/CCNPyr allowed to discriminate between a true matrix effect of Copo C_6_-H and a “solid solvent” effect (*i.e.*, simple dilution effect) on the behaviour of the phosphonated guest molecule (Figure 2).

## 2. Results and Discussion

### 2.1. Characteristics of the Host and Guest Species 

The host polymer (Copo C_6_-H) was previously synthesized by alternating cyclo-copolymerisation of *N,N*-diallyl-*N*-hexylamine and maleic acid in water, with VA086 (4 mol-%) as radical initiator [34,35,36]. The material has been characterized by elemental analysis, ^1^H and ^13^C-NMR, FT-IR, DSC, DLS and viscosimetry [34,35,36]. In the IR spectrum, the observation of C=O bands at 1717 cm^−1^ and at 1575 cm^−1^ proves that the carboxylic acid groups are present in two different forms: half of the carboxylic acids are auto-assembled in dimers via hydrogen bonds and half as carboxylate groups. Furthermore, the large band at 2560 cm^−1^ shows the presence of tertiary amine groups as ammonium functions and shows the zwitterionic structure of the polymer (Figure 1). From the X-Ray data, a 3D-lamellar arrangement of the amphiphilic macromolecules is proposed, as shown in Figure 1. This particular structure is characterized by the Bragg distance of 1.93 (±0.05) nm, calculated from the small angle diffraction line in X-Ray diffractogram (2θ < 10°). Copo C_6_-H presents a negligible intrinsic photoluminescence and its organized 3D-arrangement appears suitable to incorporate small guests (organic water-soluble or polar molecules) useful as fluorescent probes. 

The [4+2] cycloaddition of diethyl 1-phosphono-1,3-butadiene to *N*-pyrenyl maleimide (NPyrMal) gives the novel fluorescent probe which features two important substituents (Figure 2): (1) the phosphonate moiety for making hydrogen bonds with the host matrix Copo C_6_-H and for its good solubility in methanol; (2) the pyrene group for an easy detection by fluorescence spectroscopy and its sensitivity to the environment. Pyrene offers interesting photophysical properties, such as long lifetime of monomer and efficient formation of excimer by π–π stacking. These particular characteristics are used to develop various applications in biophysics [16,39], polymers [40,41,42], micelles [43,44,45] and membranes domains [46,47]. A photophysical study of this group attached or inserted to a material is a good method to provide information about the polarity of pyrene environment and the type of pyrene aggregation [48].

The photophysical data of CCNPyr in solution and in solid state have been recorded. UV/visible spectra in cyclohexane and acetonitrile present a fine structure with S_0_-S_n_ transition bands (see Supplementary information, Figure S4) and show no real influence of the medium. In both cases, the Beer-Lambert law is applicable (see Supplementary information, Figure S6). The S_0_-S_4_ band is not shifted; small bathochromic effects are observed for the S_0_-S_3_ and S_0_-S_2_ bands. Similar results were reported in the literature for 1-substituted pyrene derivatives [49]. The excimer formation is dependent of the pyrene concentration in solution (see Supplementary information, Figure S5A). The photoluminescence spectra of CCNPyr are acquired in diluted conditions in order to avoid excimer formation and to record the fine structure of the different bands. Indeed, in the absence of excimer formation (emission at 465 nm), the intensity of a few definite bands is dependent of the solvent polarity [9]. The emission spectra in cyclohexane and acetonitrile show peaks at 375 nm (I), 385 nm (III), 394 nm (V) and 415 nm (dimer) corresponding to vibronic modes of the S_1_-S_0_ emission for the S_0_-S_2_ transition (317 nm) (see Supplementary information, Figures S5A,B). In agreement with the literature data, the wavelengths of peaks are unchanged but their relative intensities are modified according to the solvent polarity. Usually, the first vibronic band (I) is dependent of the pyrene environment polarity, contrary to the third (III) and fifth (V) vibronic bands [9]; the I/III and I/V intensity ratios thus correlate with solvent polarity and can be calculated for various concentrations. Here, the I/III and I/V intensity ratios in cyclohexane are respectively 1.49 and 1.15, and in acetonitrile, a more polar solvent, they are respectively 2.31 and 1.34.

The absorption and emission spectra of CCNPyr in the solid state are depicted in the Supplementary information (Figure S7). The photoluminescence spectrum in emission mode is different from the spectrum in solution. The observed large band centered at 460 nm corresponds to the excimer formation. The fine structures of monomer bands are not present in the solid state. The Beer-Lambert law is no longer applicable, so it should be difficult to directly compare the peak intensities of “host-guest” materials and guest molecule alone because of their different concentrations in the analyzed samples.

### 2.2. Insertion of CCNPyr in Copo C_6_-H Polymeric Matrix

**Figure 3:**
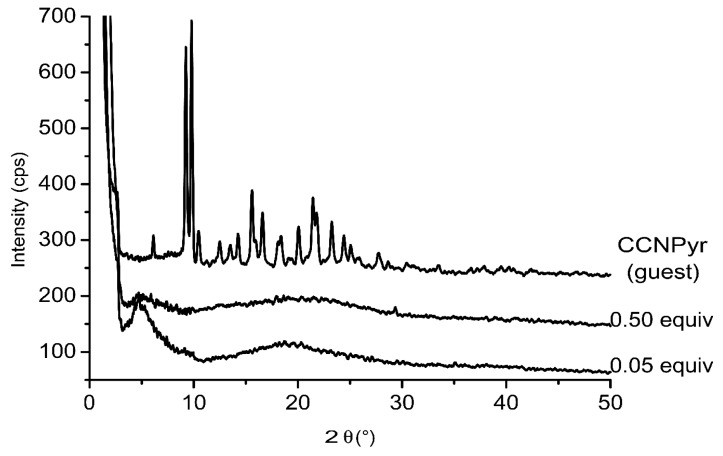
X-Ray diffractograms of guest molecule (CCNPyr) and “host-guest” materials Copo C_6_-H/CCNPyr. For sake of clarity, the diffractograms with a CCNPyr loading of 0.5 equiv. and guest molecule (CCNPyr) were shifted of + 50 cps and + 200 cps respectively. X-Ray diffractograms of other loadings are presented in Supplementary information, Figure S8.

A series of “host-guest” materials has been prepared by mixing methanolic solutions of Copo C_6_-H and CCNPyr at various concentrations. After one hour stirring under inert atmosphere, the solvent is evaporated to recover powdered solids. X-Ray diffractograms of pure CCNPyr and “host-guest” materials with different CCNPyr loadings are presented in Figure 3. The materials spectra show two zones. The first one corresponding to 2θ > 10° does not feature the fine diffraction lines of the free guest molecule. The second zone corresponding to 2θ < 10° presents one large diffraction line relative to the particular structure of the polymer matrix. The absence of diffraction lines corresponding to the CCNPyr guest for all loadings, even for the highest loading (45%wt), indicates the real homogeneity of Copo C_6_-H/CCNPyr materials and also the non-aggregation of CCNPyr probes in the Copo C_6_-H matrix. When the guest concentration increases (0.05, 0.1, 0.25 and 0.5 equiv.), the Bragg distance decreases from 1.87 nm to 1.66 nm. The pyrene groups probably interact with the alkyl chains of the polymer, leading to a packing of the 3D-structure.

**Figure 4:**
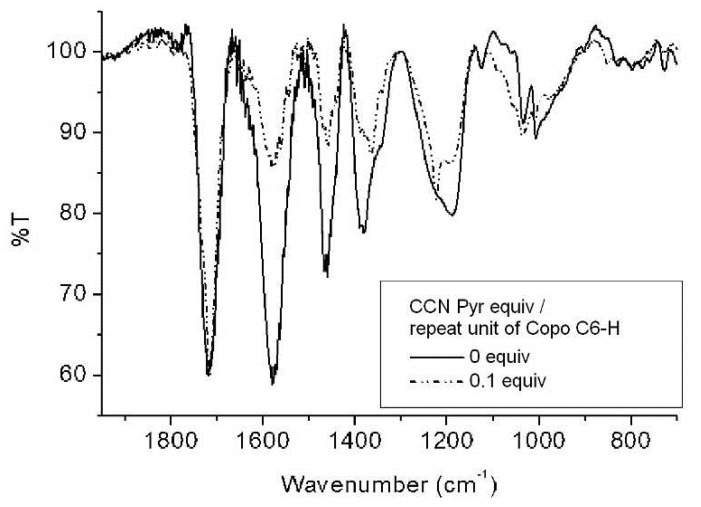
Selected IR spectra of “host-guest” materials Copo C_6_-H/CCNPyr. IR spectra of other loadings are presented in Supplementary information, Figure S9b.

The “host-guest” materials with different loadings (0.05, 0.1, 0.25 and 0.5 equiv.) are further analyzed by IR spectroscopy and compared with pure CCNPyr guest. The major CCNPyr bands (ν_C=O_ (s), ν_C=O_ (as) and ν_P=O_) superimposed with the polymer fingerprint (see Supplementary information, Figure S9a). However the shift of the phosphoryl band is well visible in the “host-guest” materials IR spectra (Figure 4) by comparison with the IR spectrum of CCNPyr (υ_P=O_ at 1245.2 cm^−1^). Copo C_6_-H/CCNPyr of 0.1 equiv. loading shows the P=O band at ν = 1222.5 cm^−1^ (Δυ_P=O_ = −22.7 cm^−1^); for 0.25 and 0.5 equiv. loadings, ν_P=O_ is observed at 1223.0 cm^−1^ (Δυ_P=O_ = −22.2 cm^−1^). These shifts suggest that the phosphoryl group interacts via hydrogen bonds with carboxylic groups and/or ammonium groups of the polymer matrix. Our P=O shifts are close to the literature value (Δυ_P=O_ = −26 cm^−1^) described in the case of diethyl phosphonate motifs involved in hydrogen bonds [50]. Only one carbonyl band is observed in the IR spectrum; ν_C=O_ COOH (host) and ν_C=O_ imide (guest) bands being superimposed, no clear conclusion could be drawn regarding the non-covalent interactions between carbonyl groups and hydrogen bonding donor/acceptor groups.

**Figure 5:**
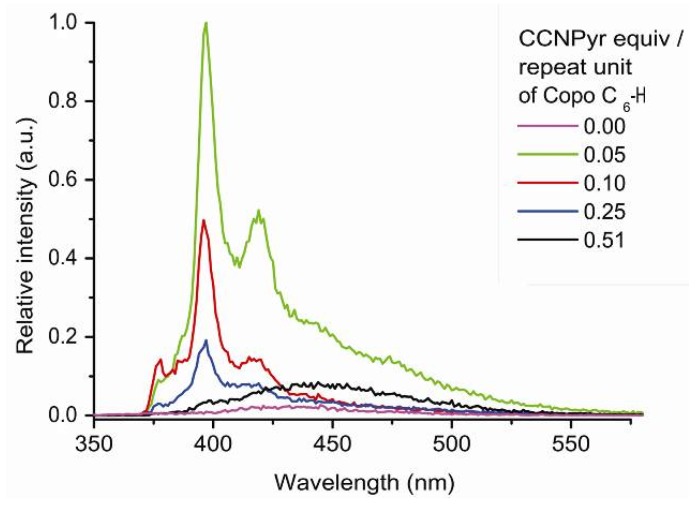
Photoluminescence spectra (emission mode, excitation at 317 nm) of “guest-host” materials Copo C_6_-H/CCNPyr in the solid state.

Next the photophysical properties of the “host-guest” materials have been recorded. The absence of excimer band and the presence of monomer and dimer bands are observed (Figure 5). The photoluminescence profile of CCNPyr in the solid state is modified by the change of environment according to the Ham effect [51]. The wavelengths of resolved vibronic bands are constant but their intensities are modified. The I/III and I/V intensity ratios are respectively equal to 0.58 and 0.22. These ratios are inferior to the ones obtained in cyclohexane solution, suggesting that the guest molecules remain into an apolar environment. The analysis of emission spectra of “host-guest” materials seems to confirm the X-Ray diffractogram results. The pyrene bands intensity increases with the diminution of the insertion ratio of guest molecule. Indeed, the pyrene photoluminescence may be quenched in the case of high CCNPyr concentration.

After redissolution of the materials in CDCl_3_ for NMR analysis, no ^31^P-NMR shift was visible, suggesting that the phosphonate group is not involved in polar interactions with the charged groups of the host matrix in the solution state (see Supplementary information, Figure S10).

### 2.3. Insertion of Pyrene (Pyr) in Copo C_6_-H Polymeric Matrix

Copo C_6_-H/Pyr “host-guest” materials have been prepared as above with 0.05, 0.1, 0.12, 0.25 and 0.5 equiv. of pyrene. X-Ray diffractograms of these materials with different loading are presented in Supplementary information (Figures S12 and S13). We have recorded two different types of diffractograms, depending on the pyrene loading, which correspond to two different types of behaviour for pyrene insertion. In the case of low loading (less than 0.25 equiv.), the diffraction line relative to the polymer matrix is only present suggesting the real insertion of pyrene guest inside the polymer matrix. At higher pyrene loading (more than 0.25 equiv.), multiple sharp lines due to the pyrene guest appear in addition of the typical polymer fingerprint; these diffractograms reveal a dilution of pyrene guest inside the polymer matrix with some pyrene aggregation, in contrast as the CCNPyr guest. The Bragg distance remains almost constant (1.74 ± 0.06 nm) upon the increase of pyrene loading. The insertion of planar pyrene molecules does not really modify the 3D structure of the matrix.

The infrared spectrum of pyrene (see Supplementary information, Figure S14a) presents multiple fine bands attributed to C-C stretching vibrations (ν_C–C_) and to C–H deformation vibration (δ_C–H_), in plane (ip) and out of plane (oop). IR spectra of Copo C_6_-H/Pyr materials reveal a shift of the δ_C-H_ ip (Δυ = −15 cm^−1^) and δ_C-H_ oop (Δυ = +12 cm^−1^) pyrene bands for the less inserted materials (loading <0.25 equiv.) while the frequency of the carbonyl bands of the matrix remains unchanged (see Supplementary information, Figure S14b and Tables S1a and S1b). The shifts of the C–H pyrene bands can be attributed to van der Waals interactions between the aromatic guest and the alkyl chain of the matrix. 

**Figure 6:**
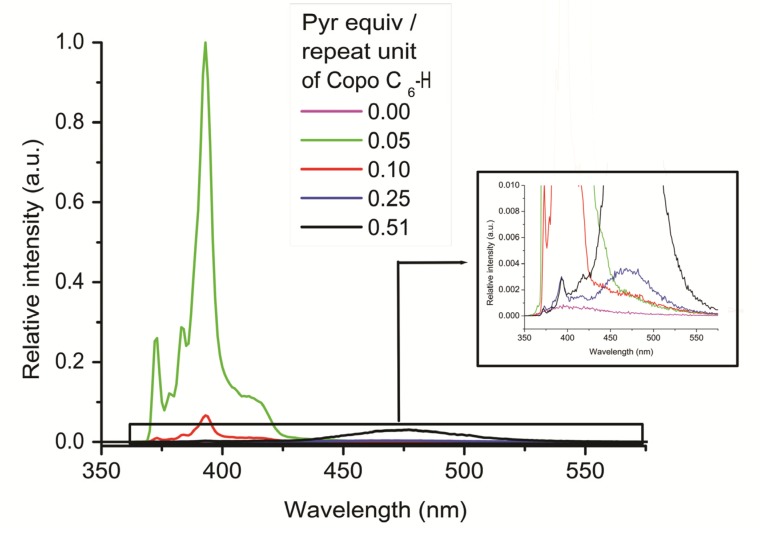
Photoluminescence spectra (emission mode, excitation at 338 nm) of “host-guest” materials Copo C_6_-H/Pyr in the solid state.

The emission spectrum of pyrene shows only one excimer band in the solid state, such as observed for CCNPyr. After insertion into Copo C_6_-H matrix, the intensity of pyrene monomer bands decreases with the enhancement of pyrene loading (Figure 6 and see Supplementary information, Figure S15), as observed for the Copo C_6_-H/CCNPyr materials (Figure 5) but this effect is more important for Copo C_6_-H/Pyr than for Copo C_6_-H/CCNPyr materials of the same loading. For example, materials with 0.25 equiv. of guests show a small contributrion of excimer band, in addition to the major contribution of monomer bands, in the case of Copo C_6_-H/CCNPyr and only excimer band for Copo C_6_-H/Pyr. We conclude that for high loadings, guest aggregates are more easily formed for Pyr than for CCNPyr, causing a more efficient quenching of the monomer emission band in the Copo C_6_-H/Pyr compared to Copo C_6_-H/CCNPyr. This is consistent with the X-Ray diffraction data showing a pyrene separate phase in Copo C_6_-H/Pyr materials with loadings above 0.25 equiv. The evolution of intensity of pyrene monomer bands of Copo C_6_-H/Pyr materials, as a function of pyrene loading, appears different from that of pyrene incorpored films made from polyethylene (PE) or polystyrene (PS), which present a “perfect solution” behavior [19,21].

By comparison with CCNPyr, the pyrene emission spectrum presents a fine structure with a well defined band at 379 nm (band II). This band is not affected by the polarity of the environment, like the third (III) and fifth (V) vibronic bands and contrary to the first band (I). The I/II, I/III and I/V intensity ratios for the Copo C_6_-H/Pyr materials, with a pyrene loading less than 0.25 equiv., are respectively equal to 1.55, 0.81 and 0.25. Considering the corresponding ratios reported in the literature for pyrene solutions [9], we can conclude that pyrene as “guest” in the Copo C_6_-H matrix belongs to a hydrophobic environment [19,21,22].

### 2.4. Insertion of N-Pyrenylmaleimide (NPyrMal) in Copo C_6_-H Polymeric Matrix

A second series of “host-guest” reference materials is similarly prepared from Copo C_6_-H and NPyrMal with the idea of detecting the possible role of carbonyl functions in the non covalent interactions. 

The X-Ray diffractograms of pure *N*-pyrenylmaleimide and “host-guest” materials with different loading are presented in Supplementary information (Figures S17 and S18). The results are very comparable to Copo C_6_-H/Pyr materials: for a loading higher than 0.25 equiv. of NPyrMal, a separate phase of “guest” appears in the “hybrid” sample; at low loading, real insertion of NPyrMal occurs. Since the first diffraction line of NPyrMal (2θ = 4.4°) is superimposed with the typical polymer matrix line, no conclusions could be clearly drawn regarding the variation of the Bragg distance. However, for NPyrMal loading less than 0.25 equiv., it seems that the Bragg distance increases (from 1.70 nm to 1.89 nm) with the NPyrMal concentration. This behaviour different from CCNPyr suggests a different localisation of NPyrMal into the Copo C_6_-H matrix.

In the IR spectra of materials with high loading, a small shift for the ν_C=O_ band of COOH (host) (Δυ = −5 cm^−1^) compared with Copo C_6_-H is observed. For materials with low loading, a very small shift of the ν_C=O_ band of imide (guest) (Δυ = +2 cm^−1^) compared with NPyrMal is visible (see Supplementary information, Figure S19 and Tables S2a and S2b). The large ν_R3NH+_ band is not shifted at all (a shift of this band is not always visible when hydrogen bonds are involved) [52]. In the literature, a negative shift has been observed for carboxylic acids (under the dimer form) interacting with pyridine groups via hydrogen bonds [53]. Various shifts have been noted for the carbonyl band of imide functions in interaction with different H-bond donor groups [54,55,56]. Our IR results are compatible with the formation of hydrogen bonds between imide carbonyl of the guest and carboxylic acid (dimer) and/or ammonium group of the host.

NPyrMal is a weak luminescent probe due to the presence of a conjugated system (*i.e.*, maleimide motif) which partially absorbs light and strongly decreases the pyrene motif fluorescence [57,58,59,60,61,62].

**Figure 7:**
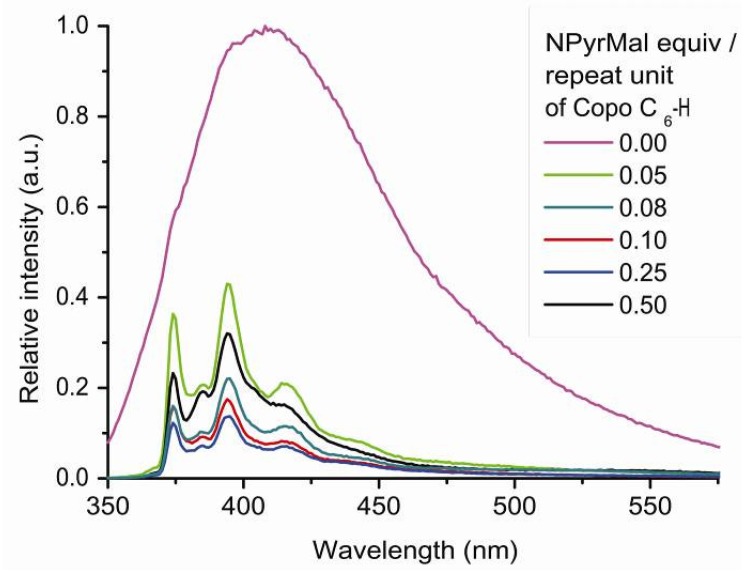
Photoluminescence spectra (emission mode, excitation at 340 nm) of “host-guest” materials Copo C_6_-H/NPyrMal in the solid state.

In solid state, the emission spectrum of NPyrMal shows only an excimer band, like Pyr and CCNPyr. After insertion into the polymer matrix, characteristic bands relative to pyrene monomer (bands I, III and V) appear in addition to excimer bands at 416 and 480 nm. The fluorescence intensity of the monomer bands increases with the decrease of NPyrMal loading, except for the 0.5 equiv. sample, which appears quite similar to the 0.05 equiv. sample (Figure 7). The I/III and I/V intensity ratios of Copo C_6_-H/NPyrMal materials, with a loading between 0.05 and 0.25 equiv., are respectively equal to 1.69 and 0.85. By comparison with Copo C_6_-H/CCNPyr materials, these ratios indicate that the pyrene motif belongs to a more polar environment in the Copo C_6_-H/NPyrMal materials (Table 1). It seems that the carbonyl groups of NPyrMal act as anchoring functions for placing the guest molecules in the hydrophilic domain of Copo C_6_-H polymer matrix.

### 2.5. Insertion of CCNPyr in Polyethylene glycol (PEG-5000) 

All the experimental results collected with the Copo C_6_-H/CCNPyr materials (see Section 2.2) strongly suggest a particular matrix effect on the guest properties, instead of a simple dilution effect. As final control experiment, we have similarly prepared and analyzed PEG-5000/CCNPyr materials. In the X-Ray diffractograms (see Supplementary information, Figure S20), the sharp lines relative to the guest molecule (CCNPyr) are present for all the tested loadings. The polymer diffractogram presents two major lines (2θ = 19.2°, d = 0.46 nm and 2θ = 23.4°, d = 0.38 nm), which are conserved in the PEG-5000/CCNPyr materials. 

**Figure 8:**
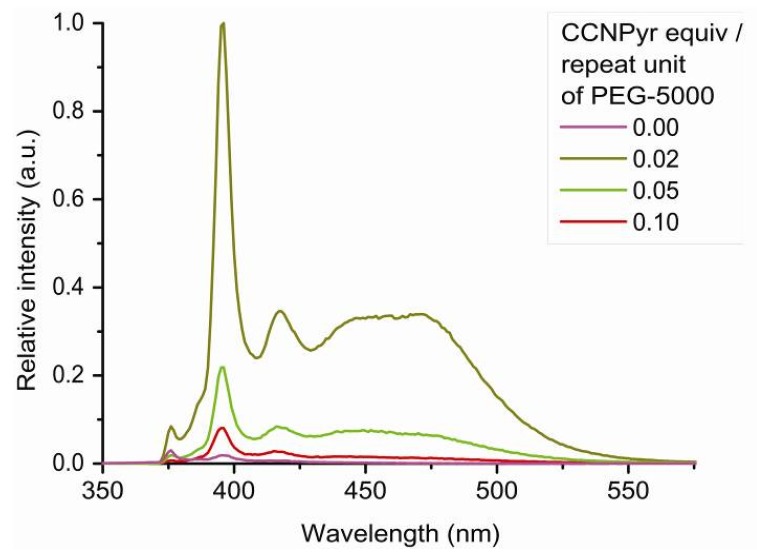
Photoluminescence spectra (emission mode, excitation at 317 nm) of “host-guest” materials PEG-5000/CCNPyr in the solid state.

The CCNPyr photoluminescence increases with the decrease of its insertion concentration, as observed with the Copo C_6_-H matrix. However, an important excimer peak is always present, even for the lowest loadings (Figure 8). The occurrence of excimers is probably due to a privileged distribution of guest molecules in amorphous regions of PEG-5000 such as observed for Pyr-PE [19], and proposed for Pyr-PMMA [24,25] and Pyr-PS films [21,22]. The excimer formation suggests a parallel arrangement of pyrene molecules with a large overlap.

**Figure 9:**
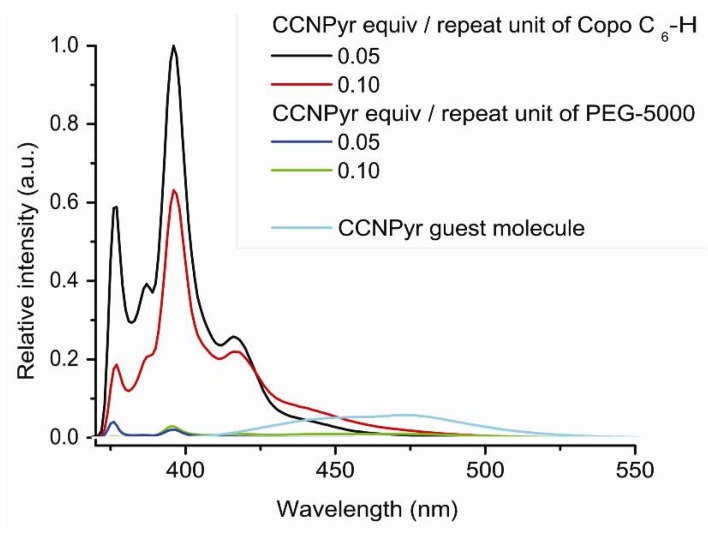
Photoluminescence spectra (emission mode, excitation at 317 nm) of “host-guest” materials Copo C_6_-H/CCNPyr and PEG-5000/CCNPyr in the solid state.

The emission intensities of Copo C_6_-H/CCNPyr materials are higher than the ones of PEG-5000/CCNPyr with the same insertion ratios (Figure 9). As an example, for a loading of 0.05 equiv., the emission of Copo C_6_-H/CCNPyr is 40 times higher than PEG-5000/CCNPyr. This observation confirms that the guest molecule is regularly dispersed into the Copo C_6_-H matrix without formation of aggregates, while in PEG-5000 matrix CCNPyr is heterogeneously located in soft domains, giving luminescence quenching under light excitation. Luminescence enhancement resulting from less polar and more rigid microenvironments has been notably described in the literature for aromatic guests inserted into cyclodextrine and calixarene cavities [37,38,63].

## 3. Experimental 

### 3.1. Materials

Polyethyleneglycol (PEG-5000) was purchased from Aldrich (Bornem, Belgium). PEG-5000 was dissolved in acetonitrile and the solvent was evaporated under reduced pressure for drying the polymer. The Copo C_6_-H polymer was prepared according to the literature [26,27]. Diethyl 1-phosphono-1,3-butadiene was synthesized using the described procedure [24,25]. Pyrene and *N*-pyrenylmaleimide were purchased from Aldrich. Methanol and acetonitrile for analysis were used as received for the preparation of “guest-host” materials. CCNPyr was prepared according to a previous procedure validated for the cycloaddition of diethyl 1-phosphono-1,3-butadiene onto a series of *N*-functionalized maleimides. [31,32,33] In all cases, the [4+2] Diels-Alder reaction led to the *endo*-diastereoisomer of cycloadduct. In the present case, due to steric factors, a 60:40 mixture of *endo*/*exo* diastereoisomers was recovered which could not be separated by column chromatography or HPLC. The NMR data showed the typical features of the bicyclic framework as previously [31,32,33], for both stereoisomers. 

### 3.2. Analytical Methods

X-ray diffraction study was carried out on a Siemens D5000 diffractometer using the Kα radiation of Cu (λ = 0.15418 nm). The 2θ range was scanned from 1 to 10° and from 10 to 70° at a rate of 0.002 and 0.01 deg·s^−1^ respectively.

Solutions for preliminary fluorescence study of guest molecule were prepared by mixing appropriate amounts of guest molecule in acetonitrile or in cyclohexane in volumetric flasks. The absorption spectra of guest molecule in solution were recorded on a Varian Cary 50 Conc UV-Vis spectrophotometer and Shimadzu UV-vis 1700 PharmaSpec spectrophotometer at room temperature using glass cells of 1 cm optical pathlength. The fluorescence measurements were recorded on two spectrophotometers: emission spectra in solid state were recorded on a Fluorolog®-3 spectrophotometer (Jobin Yvon Horiba) and emission spectra in solution were recorded on a Varian Cary Eclipse spectrophotometer using acetonitrile or cyclohexane solutions. All the measurements were performed at room temperature. The emission spectra were measured in 1 cm pathlength quartz cells, in photo counts and corrected for the instrumental function.

Infrared spectra (IR) were recorded by transmittance with a Shimadzu FTIR-8400S equipment and the absorption bands are reported in cm^−1^. Products were analysed in KBr pellets. The intensity of peaks was noted by (w), (m) and (s), respectively for weak, medium and strong.

^1^H-, ^13^C- and ^31^P-NMR spectra of CCNPyr were recorded on a Bruker AVANCE II 500 spectrometer. ^31^P-NMR spectra were recorded on a Bruker AVANCE 300 spectrophotometer operating at 121 MHz for phosphorus with complete carbon and proton decoupling (^31^P-NMR). The solutions were prepared in CDCl_3_. Chemical shifts were reported in ppm from tetramethylsilane as the internal standard (δ = 0.0 ppm) for ^1^H spectra. The internal standard was deuterated chloroform for the ^13^C spectra (δ = 77.16 ppm). ^31^P downfield shifts (δ) were expressed with a positive sign, in ppm, to external 85% H_3_PO_4_ in H_2_O. All NMR spectra were recorded at ambient temperature. Spectra were reported as follows: chemical shift (δ ppm), multiplicity (s = singlet, d = doublet, t = triplet, q = quartet, m = multiplet), coupling constants (Hz), integration, and assignment. The atom numbering (Figure 2) was not described as the IUPAC numbering. 

### 3.3. Synthesis of Pyrene-Containing Fluorescence Diethyl (1,1a,3,3a,4,7)-hexahydro-1,3-dioxo-2-(pyren-1-yl)-2H-isoindol-4-phosphonate-1,3-dione (CCNPyr, Figure 2)

A solution of diethyl 1-phosphono-1,3-butadiene (0.200 g, 1.05 mmol, 1 equiv.) and *N*-pyrenylmaleimide (0.313 g, 1.05 mmol, 1 equiv.) in xylene (2.8 mL) was stirred at 140 °C for 10 h. The reaction mixture was concentrated under vacuum and directly purified by column chromatography on silica gel (dichloromethane for removing the unreacted *N*-pyrenylmaleimide, then acetone/ethyl acetate, 1:3) to afford a brown powder (0.358 g, 69%) as a 60/40 mixture of two diastereoisomers. M.p. 97.4–101.4 °C; FTIR (Film, cm^−1^): *ν*˜ = 2982 (w), 2932 (w), 1784 (w, C=O), 1713 (s, C=O), 1602 (w), 1510 (w), 1439 (m), 1385 (m), 1244 (m, P=O), 1051 (s), 1022 (s, P–O), 966 (m, P–O), 681 cm^−1^ (w); UV-Visible (cyclohexane): λ_max_ (ε) = 244 nm (133619 L mol^−1^cm^−1^, S_4_-S_0_), 276 nm (59675 Lmol^−1^cm^−1^, S_3_-S_0_), 342 nm (73709 Lmol^−1^cm^−1^, S_2_-S_0_), (acetonitrile): λ_max_ (ε) = 244 nm (84573 Lmol^−1^cm^−1^, S_4_-S_0_), 276 nm (64246 Lmol^−1^cm^−1^, S_3_-S_0_), 342 nm (57342 Lmol^−1^cm^−1^, S_2_-S_0_); HRMS (MALDI-TOF, positive mode): *m/z* (%) calcd for C_28_H_27_NO_5_P: 488.16270 [M+H]:, found: 488.1620 (100); 460.1283 (11) [M-CH_2_=CH_2_]; 510.1381 (36) [M+Na]; 526.1135 (15) [M+K]; elemental analysis calcd (%) for C_28_H_26_NO_5_P·2H_2_O: C 64.24, H 5.78, N 2.68, P 5.92; found: C 64.67, H 5.32, N 2.35, P 5.43.

Diastereoisomer (major): *R*_f_ = 0.4 (ethyl acetate/acetonitrile 3:1); ^1^H-NMR (500 MHz, CDCl_3_) δ = 8.25 (d, *J*(H,H) = 8.1 Hz; CH pyrene), 8.22–8.18 (m; CH pyrene), 8.11 (t, *J*(H,H) = 9.5 Hz; CH pyrene), 8.08–8.08 (m; CH pyrene), 8.04–7 .98 (m; CH pyrene), 8.04–7.98 (m; CH pyrene), 7.77 (d, *J*(H,H) = 9.9 Hz; CH pyrene), 7.75 (d, *J*(H,H) = 11.0 Hz; CH pyrene), 6.25–6.20 (m, 1H; C(3)-H), 6.08 (dtd, ^3^*J*_2,3_ = 9.4 Hz, ^3^*J*_2,P_ = ^3^*J*_1,2_ = 6.4 Hz and ^4^*J*_2,4’_ = 1.9 Hz, 1H; C(2)-H), 4.14 (quint., ^3^*J*_5,6_ = ^3^*J*_5,P_ = 7.4 Hz, 4 H, C(5)-H), 3.60 (ddd, ^3^*J*_7,P_ = 20.0 Hz, ^3^*J*_7,8_ = 11.0 Hz and ^3^*J*_1,7_ = 6.4 Hz, 1H; C(7)-H), 3.55 (td, ^3^*J*_7,8_ = ^3^*J*_4’,8_ = 9.6 Hz and ^3^*J*_4,8_ = 3.1 Hz, 1H; C(8)-H), 3.35 (dt, ^1^*J*_1,P_ = 22.3 Hz, ^3^*J*_1,7_ = 6.6 Hz and ^3^*J*_1,2_ = 5.9 Hz, 1H; C(1)-H), 3.03 (ddd, ^2^*J*_4,4’_ = 16.0 Hz, ^3^*J*_4,8_ = 7.2 Hz and ^3^*J*_3,4_ = 4.8 Hz, 1H; C(4)-H), 2.91 (dddq, ^2^*J*_4,4’_ = 16.6 Hz, ^3^*J*_4’,8_ = 11.2 Hz, ^3^*J*_3,4’_ = 5.6 Hz and ^4^*J*_4’,P_ = ^4^*J*_2,4’_ = ^4^*J*_4’,7_ = 2.8 Hz, 1H; C(4)-H’), 1.30 (t, ^3^*J*_5,6_ = 6.7 Hz, 3H; C(6)-H), 1.27 (t, ^3^*J*_5’,6’_ = 7.0 Hz, 3H; C(6’)-H); ^13^C-NMR (125 MHz, CDCl_3_) δ = 179.03 (s; C(10)), 176.40 (s; C(9)), 132.08 (s; C=C pyrene), 131.02 (s; C=C pyrene), 130.59 (d, ^3^*J*_3,P_ = 11.2 Hz; C(3)), 128.88 (s; C=C pyrene), 128.68 (s; C=C pyrene), 128.49 ((s; C=C pyrene), 128.41 (s; C=C pyrene), 127.95 (s; C=C pyrene), 127.58 (s; C=C pyrene), 127.18 (s; C=C pyrene), 127.13 (s; C=C pyrene), 126.34 (s; C=C pyrene), 126.00 (s; C=C pyrene), 125.78 (s; C=C pyrene), 125.61 (s; C=C pyrene), 125.37 (s; C=C pyrene), 125.13 (s; C=C pyrene), 124.55 (s; C=C pyrene), 124.42 (s; C=C pyrene), , 124.41 (s; C=C pyrene), 121.37 (s; C=C pyrene), 125.87 (d; ^2^*J*_2,P_ =8.7 Hz; C(2)), 63.40 (d, ^2^*J*_5,P_ = 6.6 Hz; C(5)), 62.39 (d, ^2^*J*_5’,P_ = 7.2 Hz; C(5’)), 41.20 (s; C(7)), 38.98 (d, ^3^*J*_8,P_ = 5.5 Hz; C(8)), 34.31 (d, ^1^*J*_1,P_ = 141.8 Hz; C(1)), 22.29 (s; C(4)), 16.40 (d, ^3^*J*_6,P_ = 5.4 Hz; C(6)), 16.36 (d, ^3^*J*_6’,P_ = 5.7 Hz; C(6’)); ^31^P-NMR (202 MHz, CDCl_3_) δ = 25.60.

Diastereoisomer (minor): *R*_f_ = 0.3 (ethyl acetate/acetonitrile 3:1); ^1^H-NMR (500 MHz, CDCl_3_) δ = 8.25 (d, *J*(H,H) = 8.1 Hz; CH pyrene), 8.22–8.18 (m; CH pyrene), 8.11 (t, *J*(H,H) = 9.5 Hz; CH pyrene), 8.08–8.08 (m; CH pyrene), 8.04–7.98 (m; CH pyrene), 8.04–7.98 (m; CH pyrene), 7.77 (d, *J*(H,H) = 9.9 Hz; CH pyrene), 7.75 (d, *J*(H,H) = 11.0 Hz; CH pyrene), 6.38 (dtd, ^3^*J*_2,3_ = 9.5 Hz, ^3^*J*_3,4’_ = ^4^*J*_3,8_ = 4.7 Hz and ^4^*J*_3,P_ = 1.1 Hz; C(3)-H, 1H), 6.29 (dtd, ^3^*J*_2,3_ = 9.3 Hz, ^3^*J*_2,P_ = ^3^*J*_1,2_ = 5.1 Hz and ^4^*J*_2,4’_ = 1.2 Hz; C(2)-H, 1H), 4.28–4 .21 (m; C(5)-H, 2H), 4.13(q, ^3^*J*_5,6_ = 7.5 Hz; C(5’)-H’, 2H), 3.80 (ddd, ^3^*J*_7,P_ = 14.4 Hz, ^3^*J*_7,8_ = 9.7 Hz and ^4^*J*_4,7_ = 6.2 Hz; C(7)-H, 1H), 3.49 (app. td, ^3^*J*_7,8_ = 9.5 Hz, ^3^*J*_4,8_ = 8.7 Hz, ^3^*J*_4’,8_ = 4.2 Hz; C(8)-H, 1H), 3.10 (dt, ^1^*J*_1,P_ = 23.1 Hz, ^3^*J*_1,7_ = ^3^*J*_1,2_ = 5.6 Hz; C(1)-H, 1H), 2.64 (ddd, ^2^*J*_4,4’_ = 19.3 Hz, ^3^*J*_4,8_ = 9.3 Hz and ^3^*J*_3,4_ = 7.0 Hz; C(4)-H, 1H), 2.47 (dtq, ^2^*J*_4,4’_ = 15.9 Hz, ^3^*J*_4’,8_ = ^3^*J*_3,4’_ = 5.6 Hz, and ^4^*J*_4’,P_ = ^4^*J*_2,4’_ = ^4^*J*_4’,7_ = 1.3 Hz; C(4)-H’, 1H), 1.26 (t, ^3^*J*_5,6_ = 7.0 Hz; C(6)-H, 3H), 1.22 (t, ^3^*J*_5’,6’_ = 7.1 Hz; C(6’)-H, 3H); ^13^C-NMR (125 MHz, CDCl_3_) δ = 179.70 (s; C(10)), 176.12 (s; C(9)), 132.08 (s; C=C pyrene), 131.02 (s; C=C pyrene), 130.88 (d, ^3^*J*_3,P_=10.2 Hz; C(3)), 128.88 (s; C=C pyrene), 128.68 (s; C=C pyrene), 128.49 (s; C=C pyrene), 128.41 (s; C=C pyrene), 127.95 (s; C=C pyrene), 127.58 (s; C=C pyrene), 127.18 (s; C=C pyrene), 127.13 (s; C=C pyrene), 126.34 (s; C=C pyrene), 126.00 (s; C=C pyrene), 125.78 (s; C=C pyrene), 125.61 (s; C=C pyrene,, 125.37 (s; C=C pyrene), 125.13 (s; C=C pyrene), 124.55 (s; C=C pyrene), 124.42 (s; C=C pyrene), 124.41 (s; C=C pyrene), 123.54 (d, ^2^*J*_2,P_ = 9.3 Hz; C(2)), 121.37 (s; C=C pyrene), 63.51 (d, ^2^*J*_5,P_ = 6.6 Hz; C(5)), 62.03 (d, ^2^*J*_5’,P_ = 7.0 Hz; C(5’)), 41.66 (d, ^2^*J*_7,P_ = 3.1 Hz; C(7)), 40.29 (d, ^3^*J*_8,P_ = 5.3 Hz; C(8)), 34.57 (d, ^1^*J*_1,P_ = 149.7 Hz; C(1)), 24.01 (s; C(4)), 16.50 (d, ^3^*J*_6,P_ = 5.9 Hz; C(6)); ^31^P-NMR (202 MHz, CDCl_3_) δ = 25.87.

### 3.4. General Procedure for the Preparation of “Host-Guest” Materials

The polymer matrix and guest molecules were dried under high vacuum for removing traces of moisture. A polymer solution (Copo C_6_-H or PEG-5000) with a concentration of 1 mg/mL in methanol was prepared at room temperature, by sonication (20 min) in an ultrasound bath. A mother solution of guest molecule (CCNPyr in methanol, or pyrene or *N*-pyrenylmaleimide in acetonitrile) was prepared at a concentration of 0.5 equiv. of guest per repeat unit of polymer. The solution was stirred during one hour under argon atmosphere at room temperature. Daughter solutions of guest molecule were made by successive dilutions of the mother solution with the appropriate solvent (methanol for CCCNPyr guest or acetonitrile for pyrene or *N*-pyrenylmaleimide guests). The daughter solutions were stirred at room temperature during 15 min under argon atmosphere. The solution of polymer was added to the solution of guest molecule with the desired amount of equivalents of guest molecule per repeat unit of polymer. The homogeneous mixture was stirred at room temperature during one hour under argon atmosphere. The solvent was evaporated under reduced pressure (bath temperature 38 °C). “Guest-host” materials were obtained after complete evaporation of the solvent, as powdered solids (details given in Table 2). These samples were studied by powder X-Ray diffraction, IR spectroscopy and photoluminescence (in the solid state). The total removal of solvent was controlled by solution NMR of an aliquot.

## 4. Conclusions 

**Figure 10:**
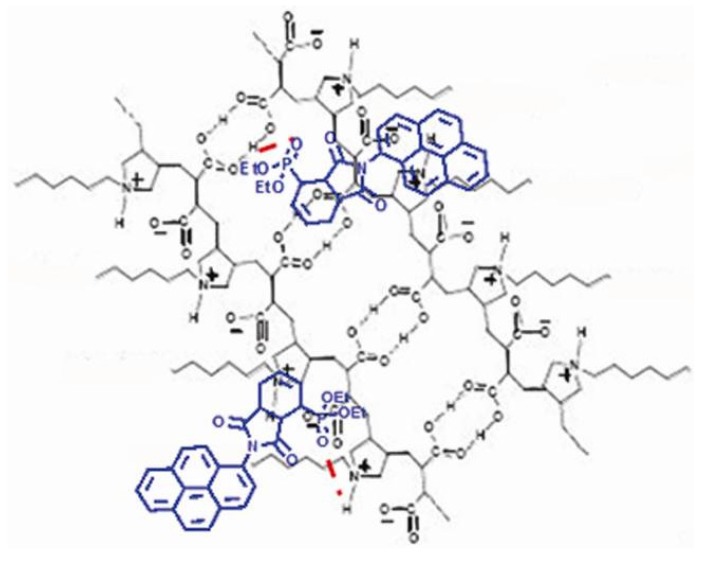
Schematic representation of the postulated non covalent interactions, in the solid state, between CCNPyr and the Copo C_6_-H matrix.

Pyrene derivatives are known as useful fluorescent probes for monitoring the polarity of materials. In this article, we have disclosed a pyrene-containing probe (CCNPyr) having a diethyl phosphonate group and illustrated the use of this probe for the elaboration of “host-guest” materials with a 3D-structurated amphiphilic copolymer (Copo C_6_-H). CCNPyr can be readily inserted into Copo C_6_-H to give homogeneous materials with a good dispersion of the guest molecules for a range of loadings from 0.05 to 0.5 equiv. (7.6 wt% to 45 wt%). The supramolecular structure of the polymer matrix is preserved (XRD data), the guest phosphonate group makes H-bonds within the host polar domain (IR data), and the pyrene group is lying in the host alkyl chain domain without aggregate formation (luminescence data). ”Host-guest” materials similarly prepared from pyrene (Pyr) show a non-homogeneous dispersion of pyrene (above 0.25 equiv.; 14.3 wt%), forming aggregates in the alkyl chain domain of Copo C_6_-H. A different behavior is observed with *N*-pyrenyl maleimide (NPyrMal) which is buried in the polar domain of Copo C_6_-H. Our vision is that the phosphonate function of CCNPyr behaves as a powerful anchorage motif targeting the H-bond donors (carboxylic acids/ammonium groups) domain of the amphiphilic copolymer matrix. As a result of the distance (about 0.93 nm) between the phosphonate head and the pyrenyl tail of CCNPyr, the pyrene probe is inevitably located in the hydrophobic domain of Copo C_6_-H (Figure 10).

The comparaison of PEG-5000 and Copo C_6_-H as “host” for the insertion of CCNPyr confirms the particular matrix effect of Copo C_6_-H. For all the range of loadings, PEG-5000/CCNPyr materials show separate phases (XRD data) with CCNPyr aggregates formed most probably in the amorphous regions of the semi-crystalline PEG-5000 polymer (luminescence data) which behaves simply as a solid solvent.

In conclusion, the fluorescent probe CCNPyr appears suitable to analyze a solid matrix featuring micro-domains of different polarities. Since the phosphonate group, in cooperation with the neighbouring carbonyl function, has been recently demonstrated to form coordination complexes with di- and trivalent metal cations [31,32,33], applications of CCNPyr could be expected for the analysis of organic-inorganic hybrid materials in the solid state.

## Figures and Tables

**Table 1:** Intensity ratios of pyrene bands in photoluminescence spectra.

Entry	Material	I/II	I/III	I/V
1	CCNPyr in cyclohexane	/	1.49	1.15
2	CCNPyr in acetonitrile	/	2.31	1.34
3	Copo C_6_-H/CCNPyr	/	0.58	0.22
4	Copo C_6_-H/Pyr (≤0.25 equiv.)	1.55	0.81	0.25
5	Copo C_6_-H/NPyrMal (≤0.25 equiv.)	/	1.69	0.85

**Table 2:** Mass of guest for each “host-guest” material and corresponding %wt.

Guest loading (equiv.)	m CCNPyr (mg)	% wt	m Pyr (mg)	% wt	m NPyrMal (mg)	% wt	m CCNPyr (mg)	% wt
	for 200 mg of Copo C_6_-H						for 200 mg of PEG-5000	
0.03	/	/	/	/	/	/	66.4	24.9
0.05	16.4	7.6	6.7	3.2	9.9	4.7	110.7	35.6
0.08	/	/	/	/	15.9	7.4	/	/
0.10	32.8	14.1	13.3	6.2	19.9	9.0	221.3	52.5
0.12	/	/	/	7.4	/	/	/	/
0.25	81.9	29.1	33.3	14.3	49.7	19.9	/	/
0.50 or 0.51	163.8	45.0	68.0	25.4	99.3	33.2	/	/

## Supplementary Materials

^1^H, ^13^C, ^31^P-NMR spectra of the guest molecule (CCNPyr), X-Ray data, ^31^P-NMR, FT-IR and photoluminescence spectra of “host-guest” materials are presented in the Supplementary Materials.

Supplementary materials can be accessed at:  http://www.mdpi.com/1420-3049/18/2/1897/s1.
