# Supplementary material for: A Pyrene- and Phosphonate-Containing Fluorescent Probe as Guest Molecule in a Host Polymer Matrix
Source: Molecules. 2013 Feb 1;18(2):1897–915. doi: 10.3390/molecules18021897 (PMC6270169; doi:10.3390/molecules18021897)
Supplement: Supplementary file 1 [file molecules-18-01897-s001.pdf]

Supporting Information

Content

I. CCNPyr guest molecule S2

I.1. NMR spectra S2

I.2. Photoluminescence data S5

II. "Host-guest" materials Copo C_6-H/CCNPyr S7

II.1. X-Ray data S7

II.2. FT-IR spectra S8

II.3. ^{31}P-NMR spectra S9

III. "Host-guest" materials Copo C_6-H/Pyr S10

III.1. X-Ray data S10

III.2. FT-IR spectra S11

III.3. Photoluminescence data S13

IV. "Host-guest" materials Copo C_6-H/NPyrMal S14

IV.1. X-Ray data S14

IV.2. FT-IR spectra S14

V. "Host-guest" materials PEG-5000/CCNPyr S17

V.1. X-Ray data S17

I. CCNPyr Guest Molecule

I.1. NMR Spectra

^{1}H-NMR spectrum

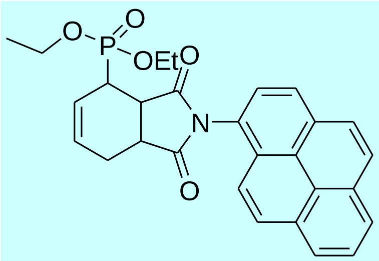

Figure S1. ^{1}H-NMR spectrum of CCNPyr ($CDCl_3$, room temperature).

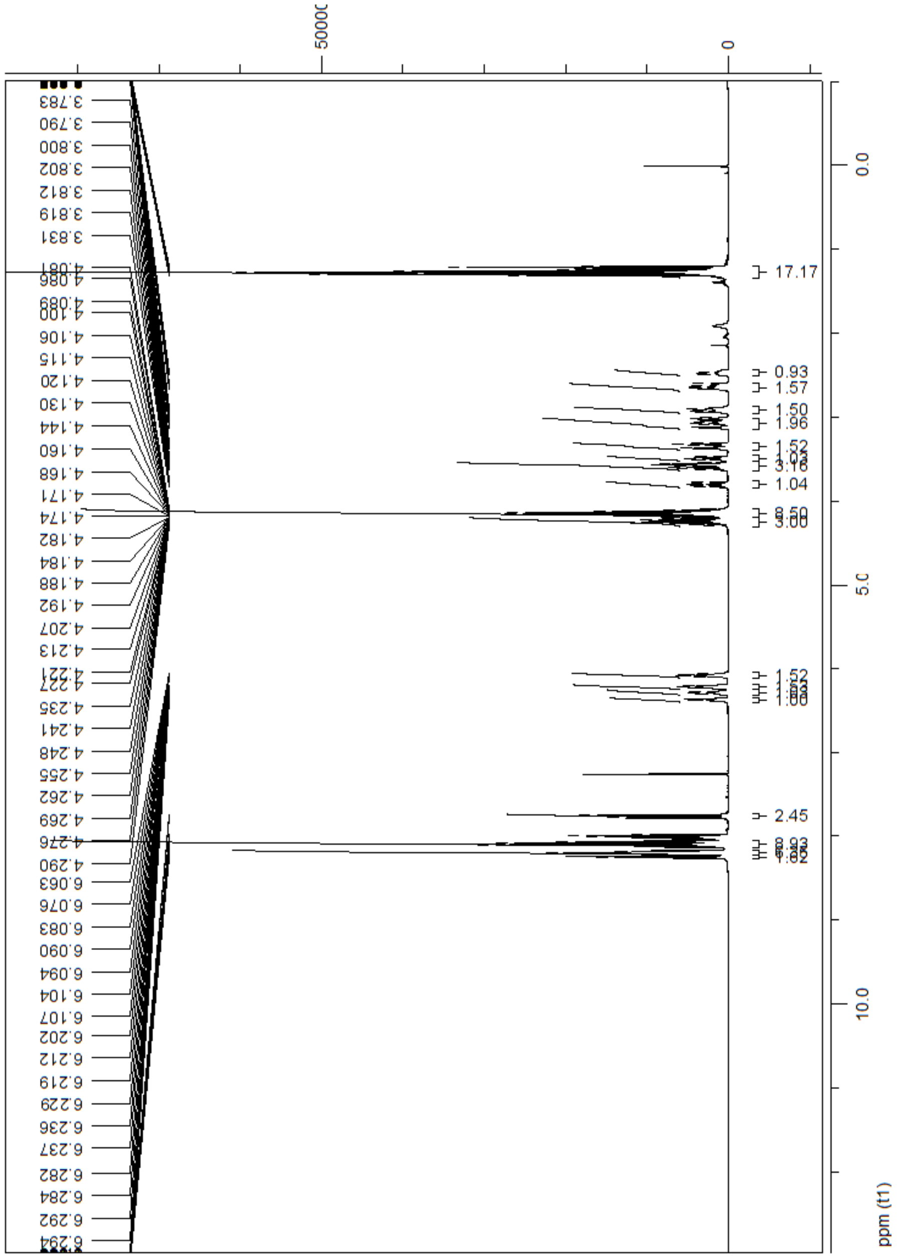

^{13}C-NMR spectrum

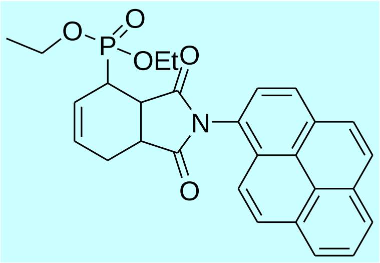

Figure S2. ^{13}C-NMR spectrum of CCNPyr ($CDCl_3$, room temperature).

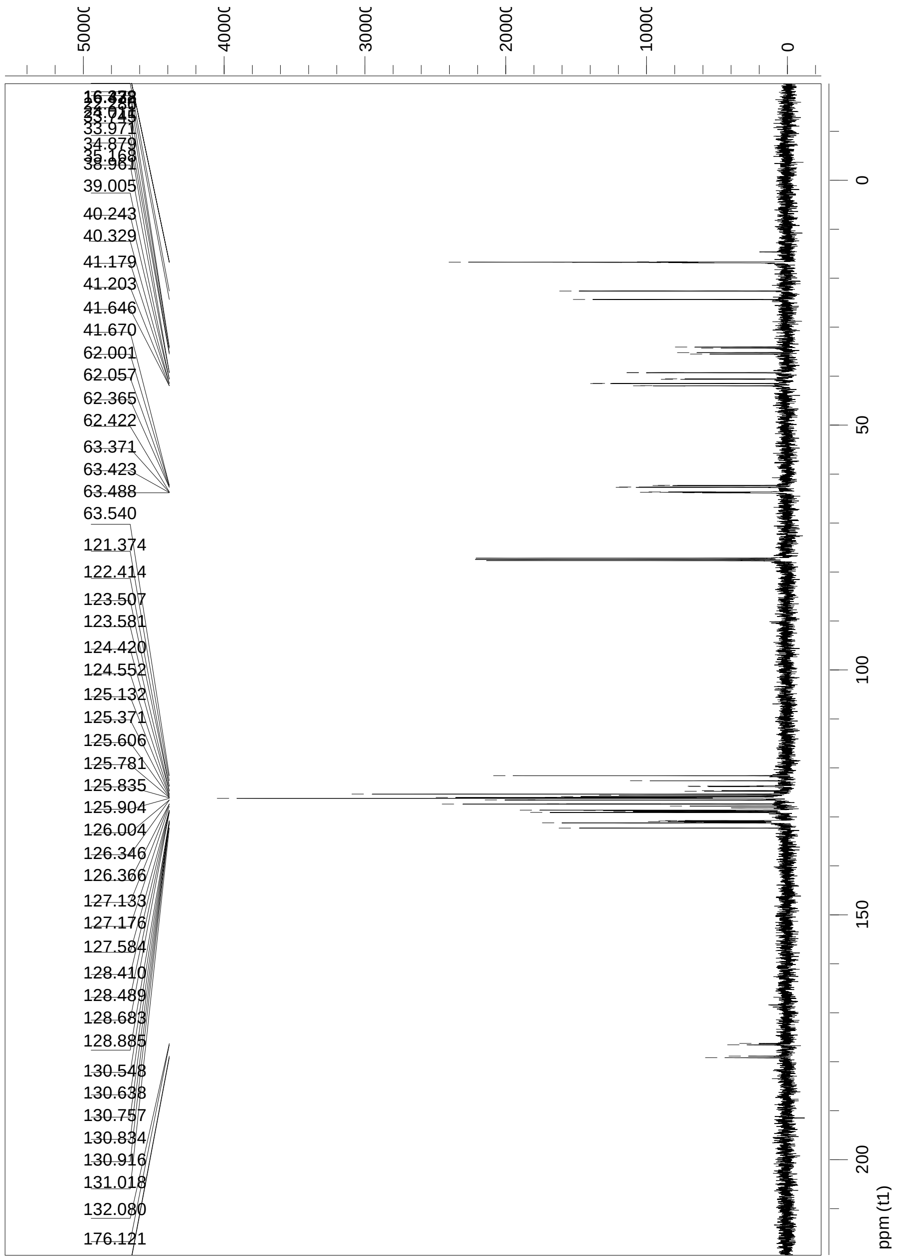

^{31}P-NMR spectrum

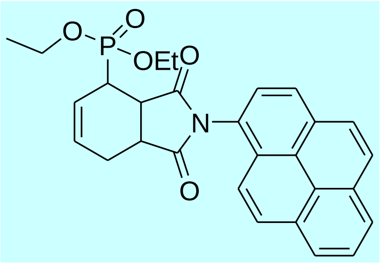

Figure S3. ^{31}P-NMR spectrum of CCNPyr ($CDCl_3$, room temperature, 85 % H_3PO_4 external probe).

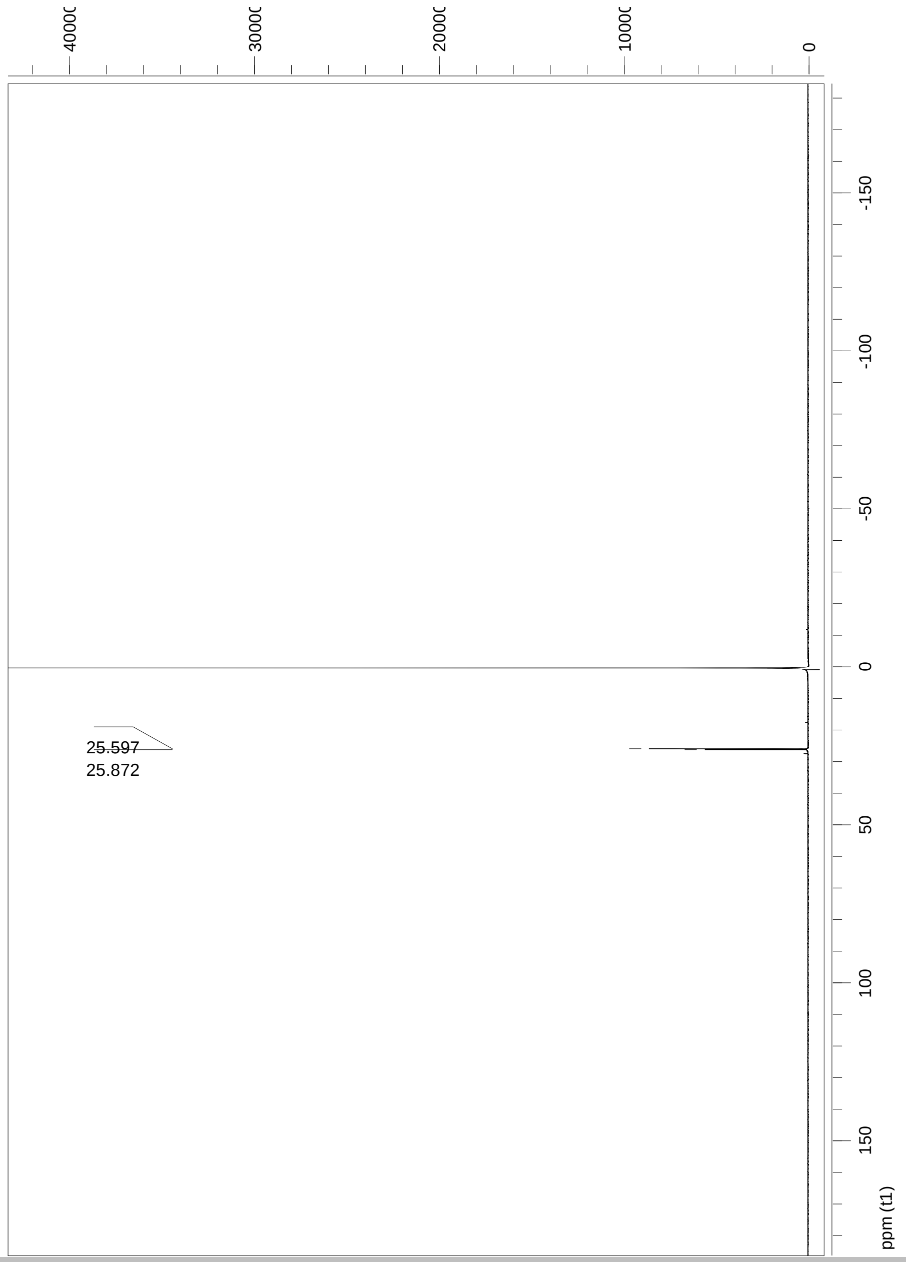

I.2. Photoluminescence Data

Figure S4. Absorption spectra of CCNPyr at various concentrations in two solvents (cyclohexane [CCNPyr] from $5.03.10^{-6}$ M to $2.45.10^{-9}$ M or acetonitrile [CCNPyr] from $4.41.10^{-6}$ M to $2.15.10^{-9}$ M).

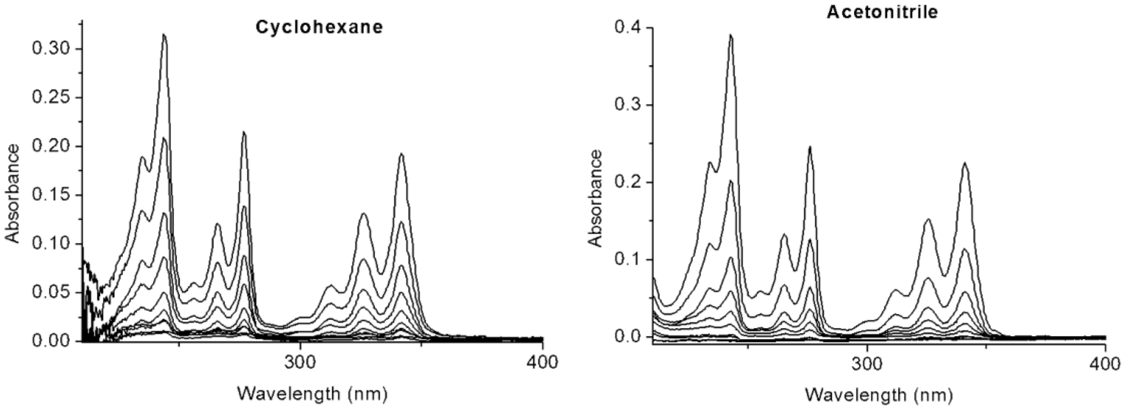

Figure S5. (**A**) Photoluminescence spectra (emission mode, excitation at 317 nm) of CCNPyr at various concentrations in cyclohexane ([CCNPyr] from $1.26.10^{-6}$ M to $1.23.10^{-9}$ M) and acetonitrile ([CCNPyr] from $2.21.10^{-6}$ M to $2.15.10^{-9}$ M); (**B**) Photoluminescence spectra (emission mode, excitation at 317 nm) of CCNPyr at almost-similar concentration in both solvents.

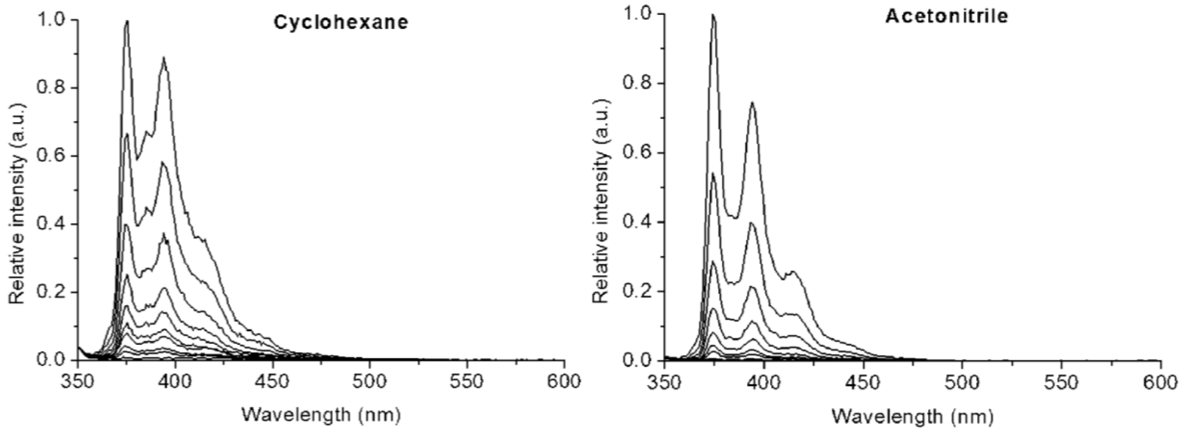

(**A**)

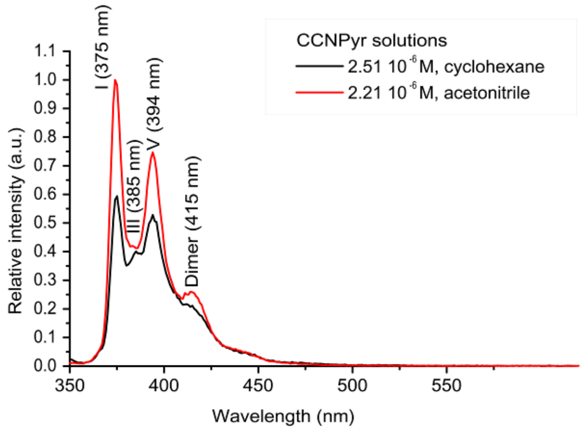

(**B**)

Figure S6. Beer-Lambert graph for solutions of CCNPyr at various concentrations in cyclohexane and acetonitrile.

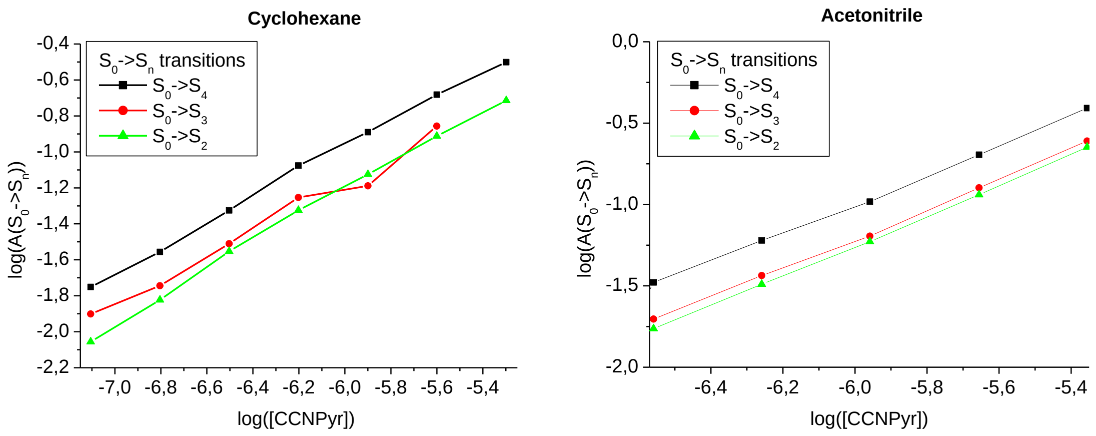

Figure S7. (**a**) Absorption spectrum of CCNpyr in the solid state; (**b**) Photoluminescence spectrum (emission mode, excitation at 317 nm) of CCNPyr in the solid state.

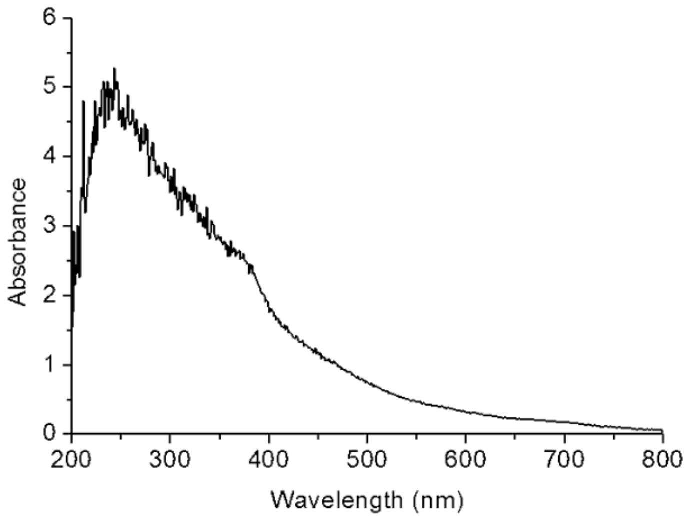

(**a**)

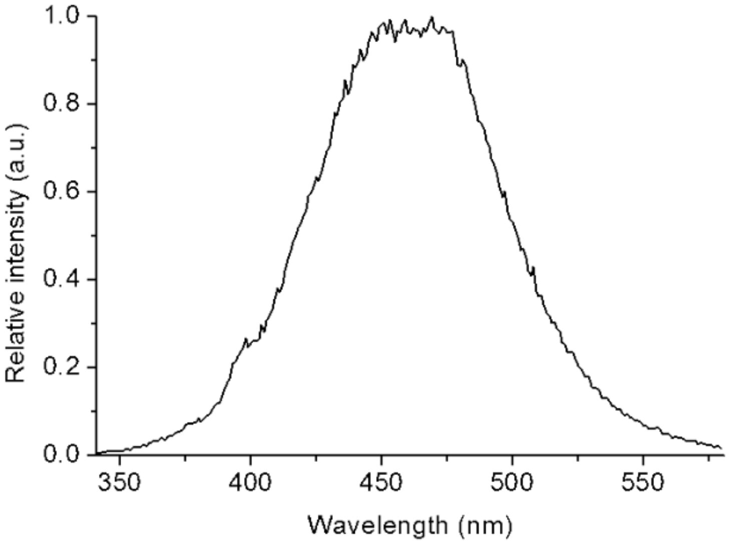

(**b**)

II. "Host-guest" materials Copo C_6-H/CCNPyr

II.1. X-Ray data

Figure S8. X-Ray diffractograms of "host-guest" materials Copo C_6-H/CCNPyr. For sake of clarity, each diffractogram with a positive CCNPyr loading was shifted of + 100 cps one of the other.

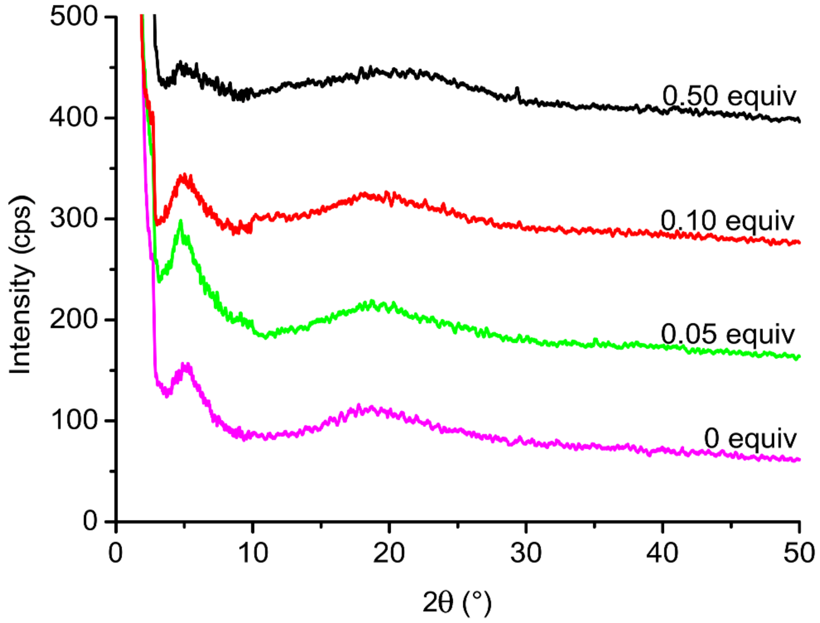

II.2. FT-IR spectra

Figure S9. (**a**) IR spectra of CCNPyr (guest) and Copo C_6-H matrix (host); (**b**) IR spectra of "host-guest" materials Copo C_6-H/CCNPyr.

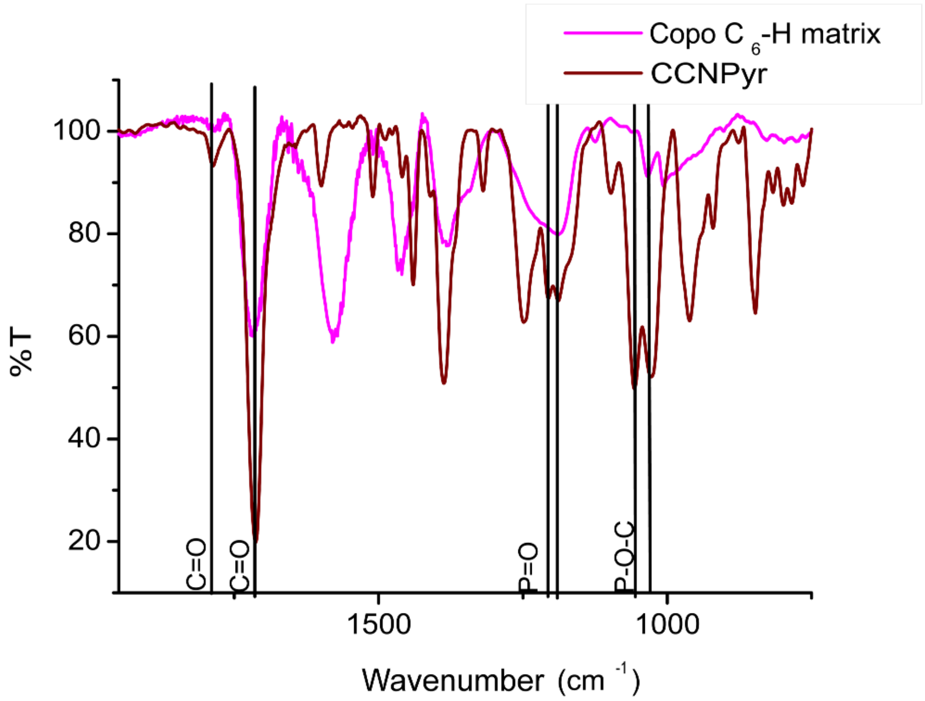

(**a**)

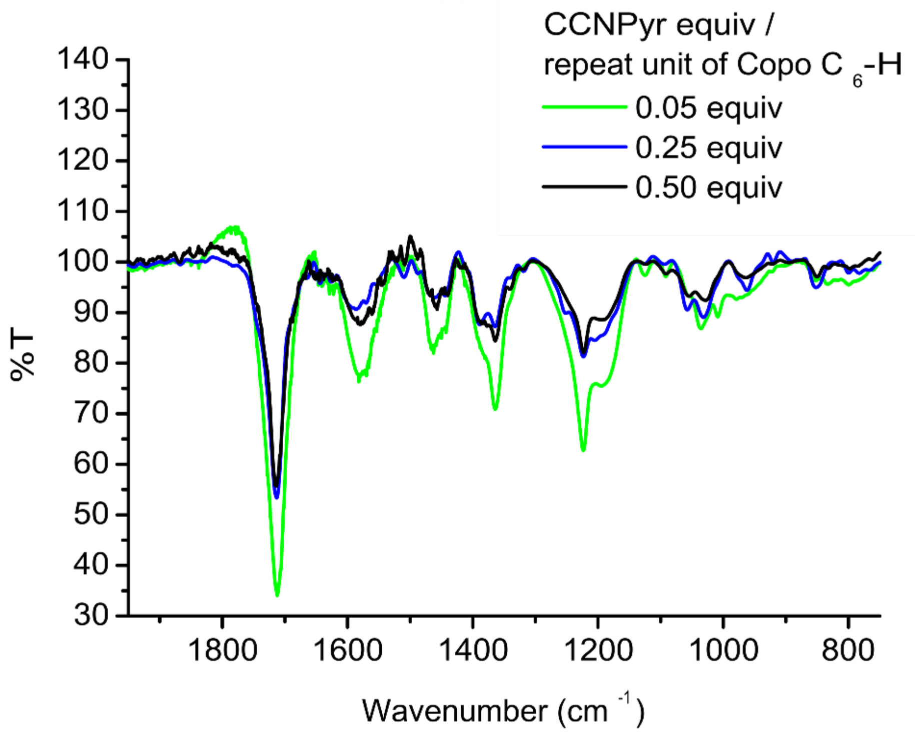

(**b**)

II.3. ^{31}P-NMR spectra

Figure S10. ^{31}P-NMR spectra of Copo C_6-H/CCNPyr redissolved in $CDCl_3$.

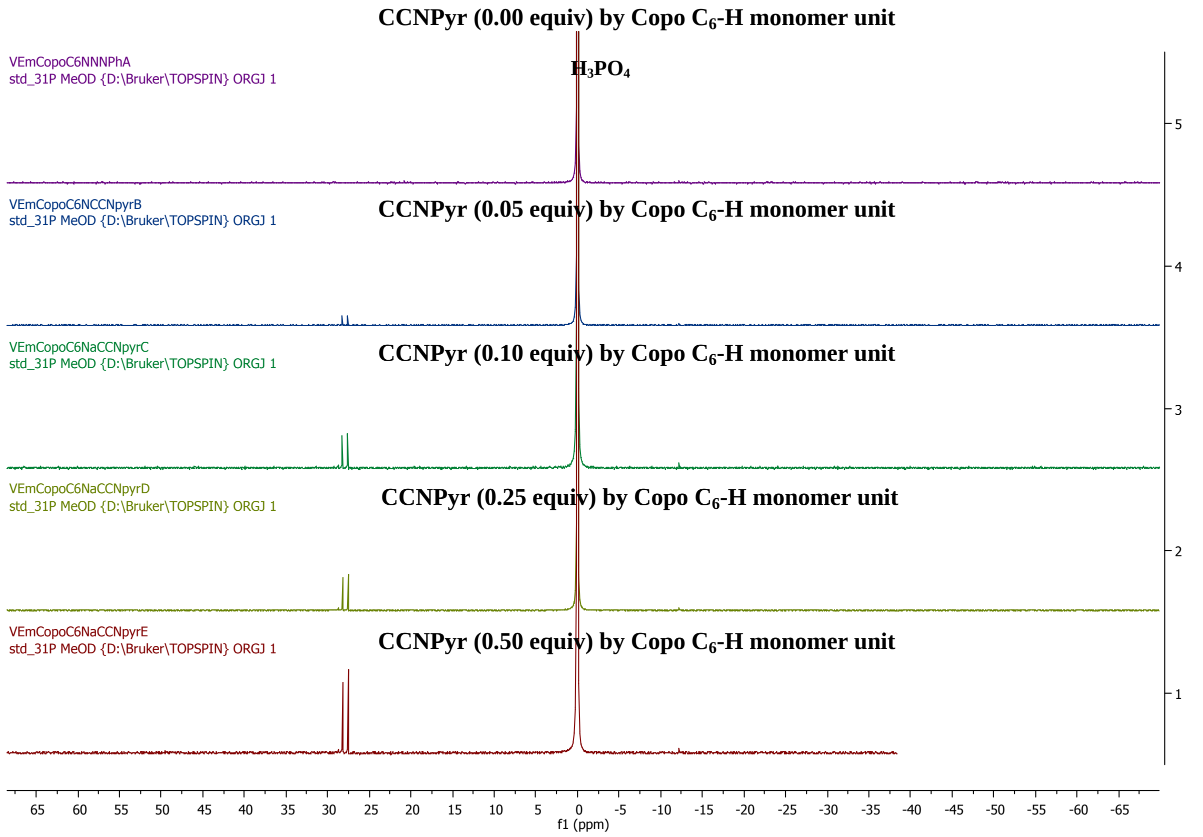

III. "Host-guest" materials Copo C_6-H/Pyr

III.1. X-Ray data

Figure S11. Experimental X-Ray diffractogram of pyrene.

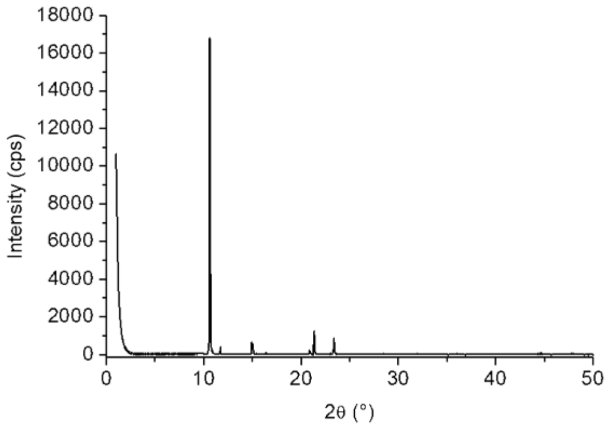

Figure S12. Theoretical X-Ray diffractogram of pyrene. (calculated with the X-Ray data described in "R.Allmann *Z.Kristallogr.,Kristallgeom.,Kristallphys., Kristallchem.* **1970**, *132*, 129").

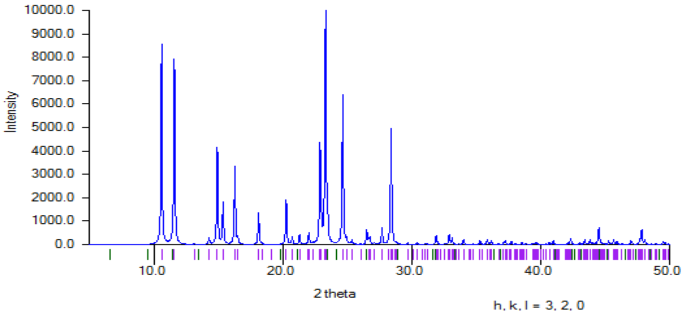

Figure S13. X-Ray diffractograms of "host-guest" materials Copo C_6-H/Pyr. For sake of clarity, each diffractogram with a positive Pyr loading was shifted of + 300 cps one of the other.

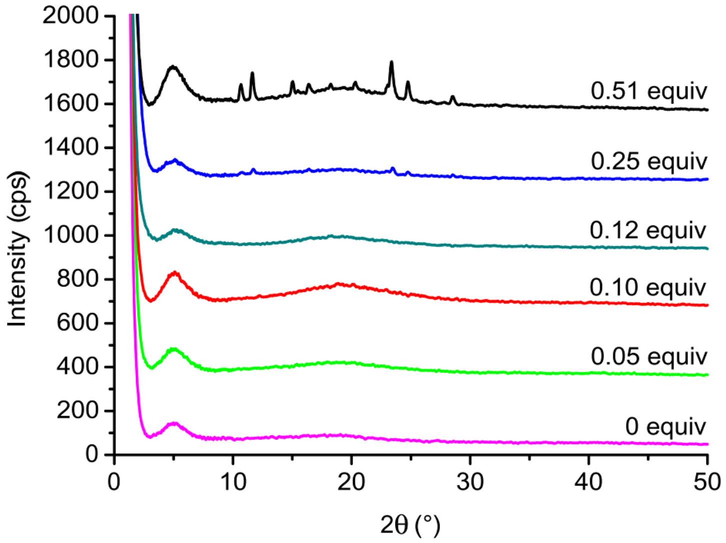

III.2. FT-IR spectra

Figure S14. (**a**) IR spectra of Pyr (guest) and Copo C_6-H matrix (host); (**b**) IR spectra of "host-guest" materials Copo C_6-H/Pyr.

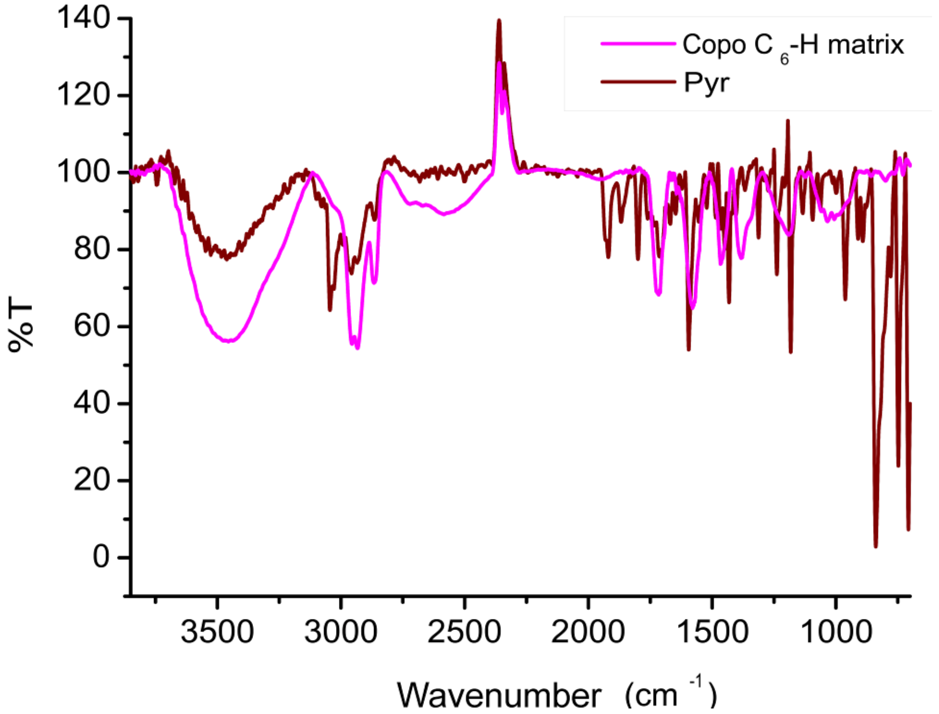

(**a**)

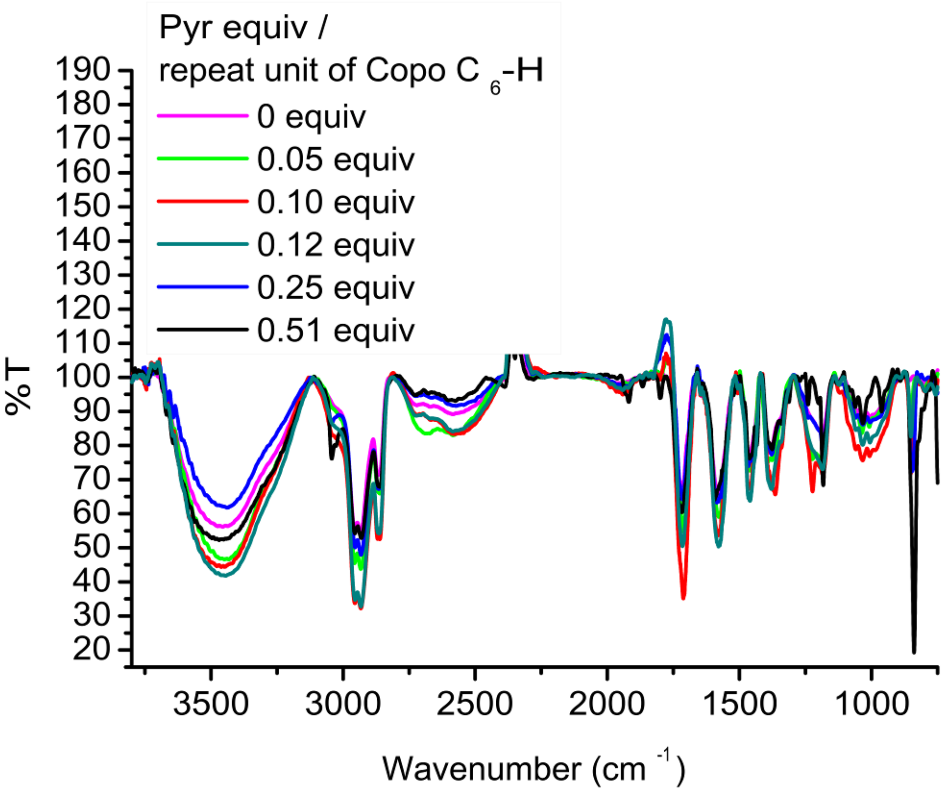

(**b**)

Table S1a. IR data for the $\delta_{C\text{-}H}$ ip bands for pyrene and Copo C_6-H/Pyr materials.

		**ῡ (cm^{-1})**	**Δῡ**	**ῡ (cm^{-1})**	**Δῡ**
Pyrene (guest)		1239	/	1182	/
Materials (Pyr equiv)	0.05	1224	-15	1188	+6
	0.1	1224	-15	1185	+3
	0.12	/	/	1186	+4
	0.25	1241	+2	1184	+2
	0.51	1240	+1 [a]	1183	+1 [a]

[a] Not significant shift (Resolution = 0.8 cm^{-1}).

Table S1b. IR data for the $\delta_{C\text{-}H}$ oop bands for pyrene and Copo C_6-H/Pyr materials.

		**ῡ (cm^{-1})**	**Δῡ**	**ῡ (cm^{-1})**	**Δῡ**	**ῡ (cm^{-1})**	**Δῡ**
Pyrene (guest)		839	/	748	/	707	/
Materials (Pyr equiv)	0.05	851	+12	757	+9	715	+8
	0.1	851	+12	757	+9	715	+8
	0.12	851	+12	757	+9	715	+8
	0.25	846	+7	752	+4	712	+5
	0.51	839	0	749	+1 [a]	709	+2

[a] Not significant shift (Resolution = 0.8 cm^{-1}).

III.3. Photoluminescence data

Figure S15. Photoluminescence spectra (emission mode, excitation at 317 nm) of "guest-host" materials Copo C_6-H/Pyr. in the solid state.

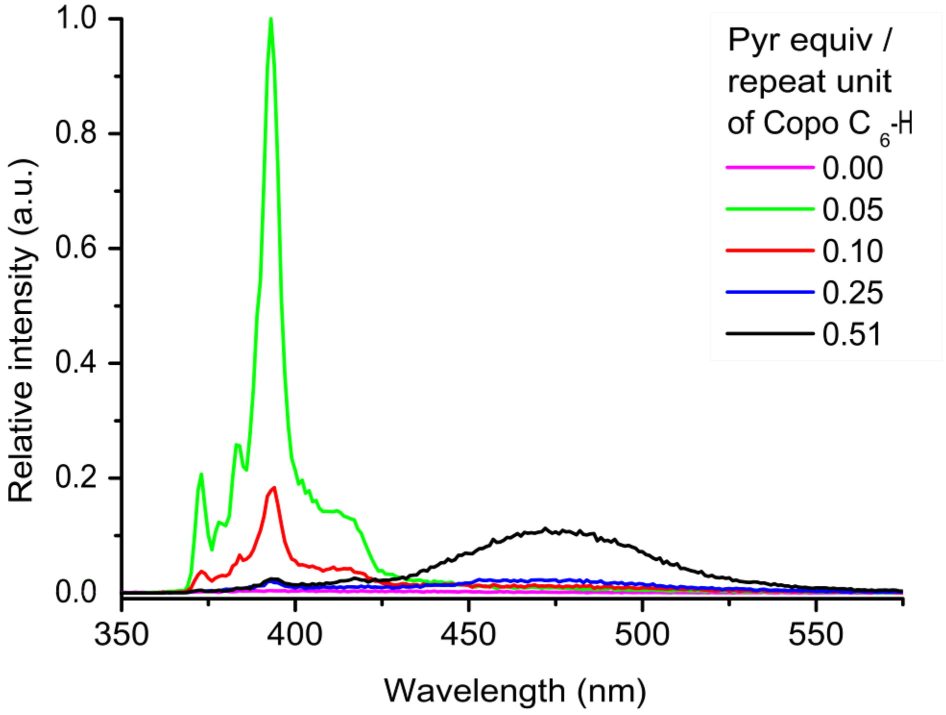

Figure S16. Comparison of I_x/I_y emission intensity ratios with the values of pyrene (from the litterature: Kalyanasundaram, K.; Thomas, J.K. *J. Am. Chem. Soc.* **1977**, *99*, 2039–2044).

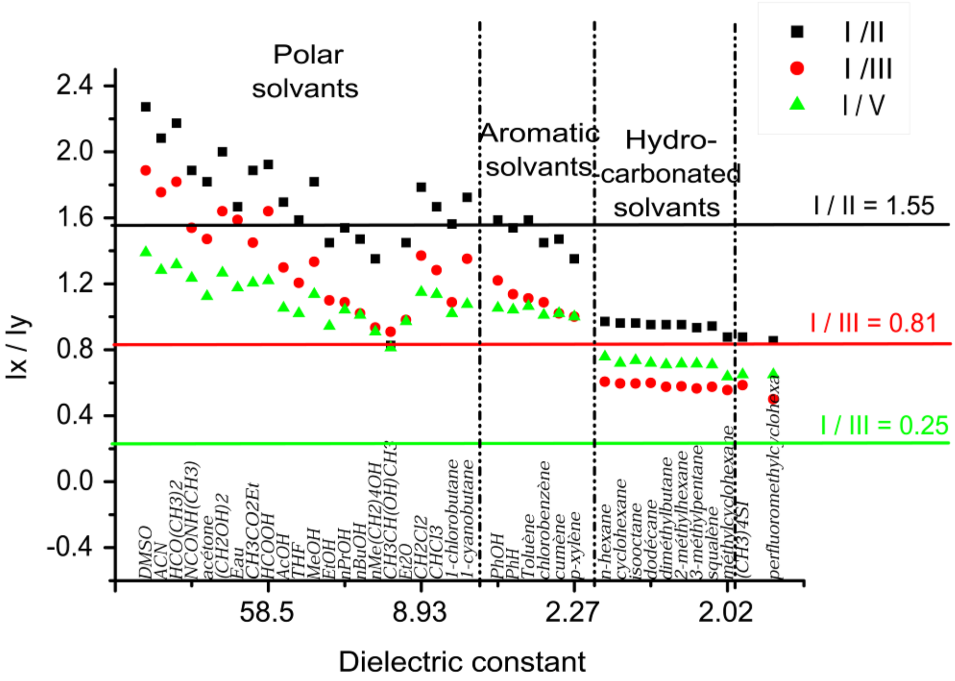

IV. "Host-guest" materials Copo C_6-H/NPyrMal

IV.1. X-Ray data

Figure S17. X-Ray diffractogram of *N*-pyrenyl maleimide.

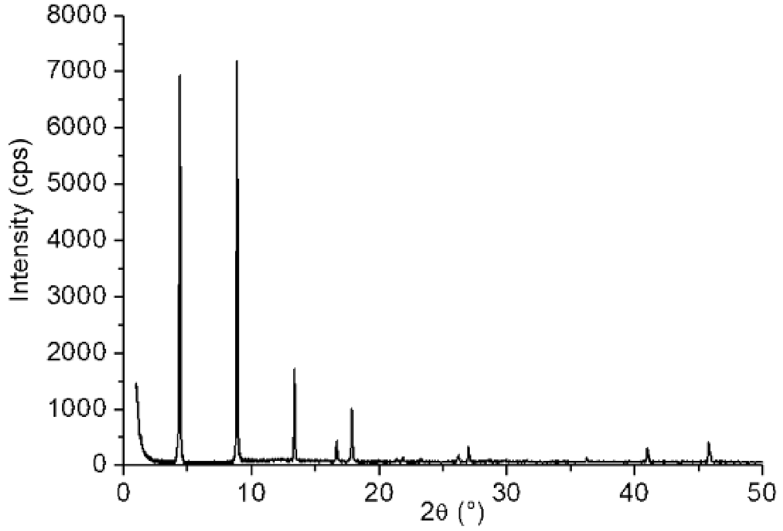

Figure S18. X-Ray diffractograms of "host-guest" materials Copo C_6-H/NPyrMal for sake of clarity, each diffractogram with a positive NPyrMal loading was shifted of + 400 cps one of the other.

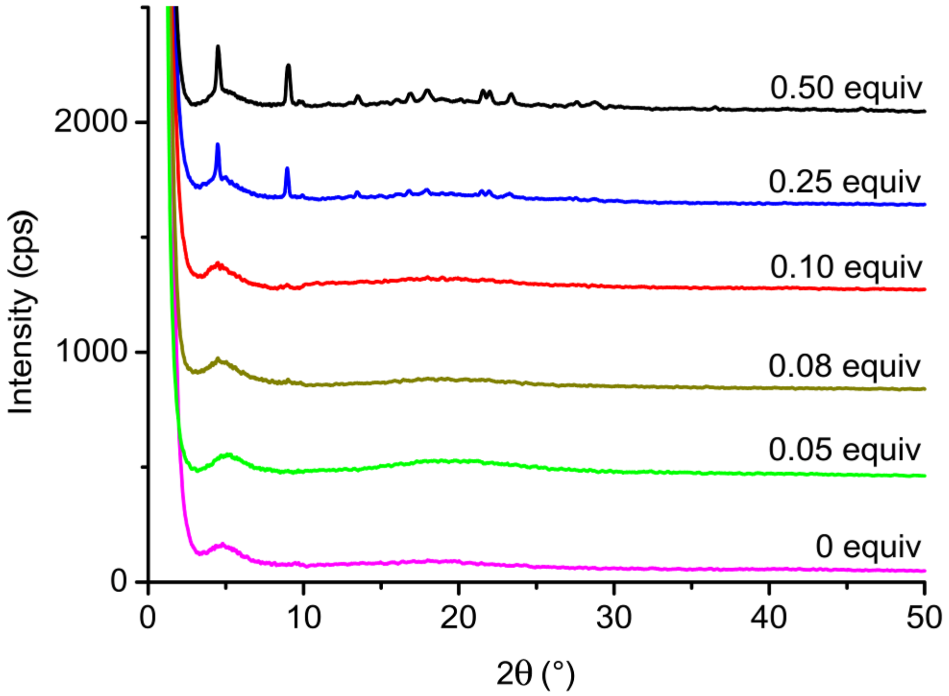

IV.2. FT-IR spectra

Figure S19. (**a**) IR spectra of NPyrMal (guest) and Copo C_6-H matrix (host); (**b**) IR spectra of "host-guest" materials Copo C_6-H/NPyrMal.

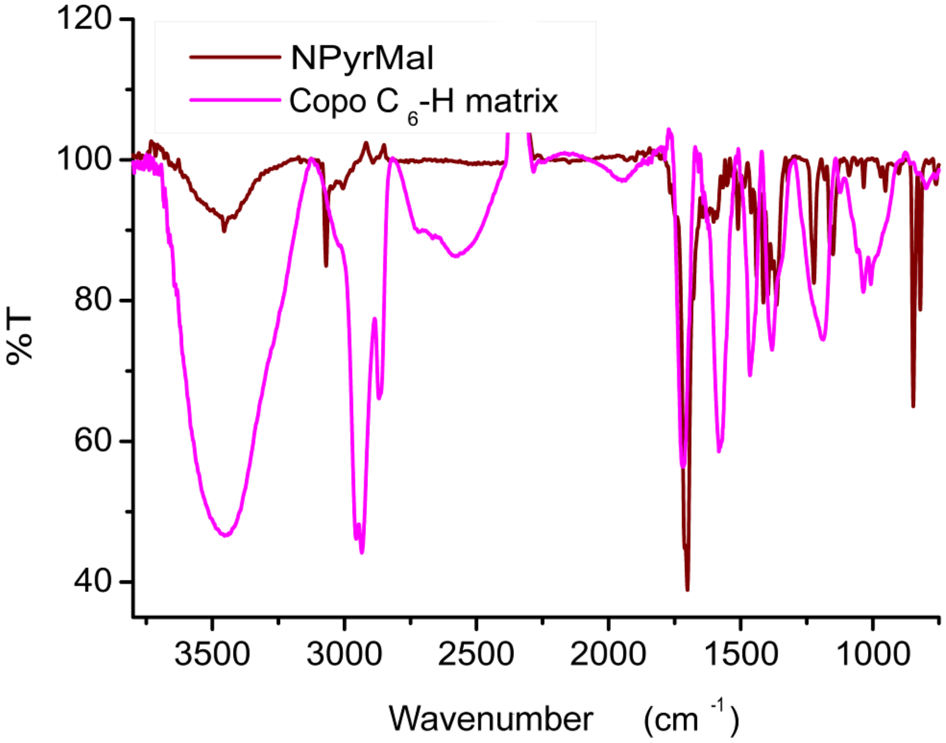

(**a**)

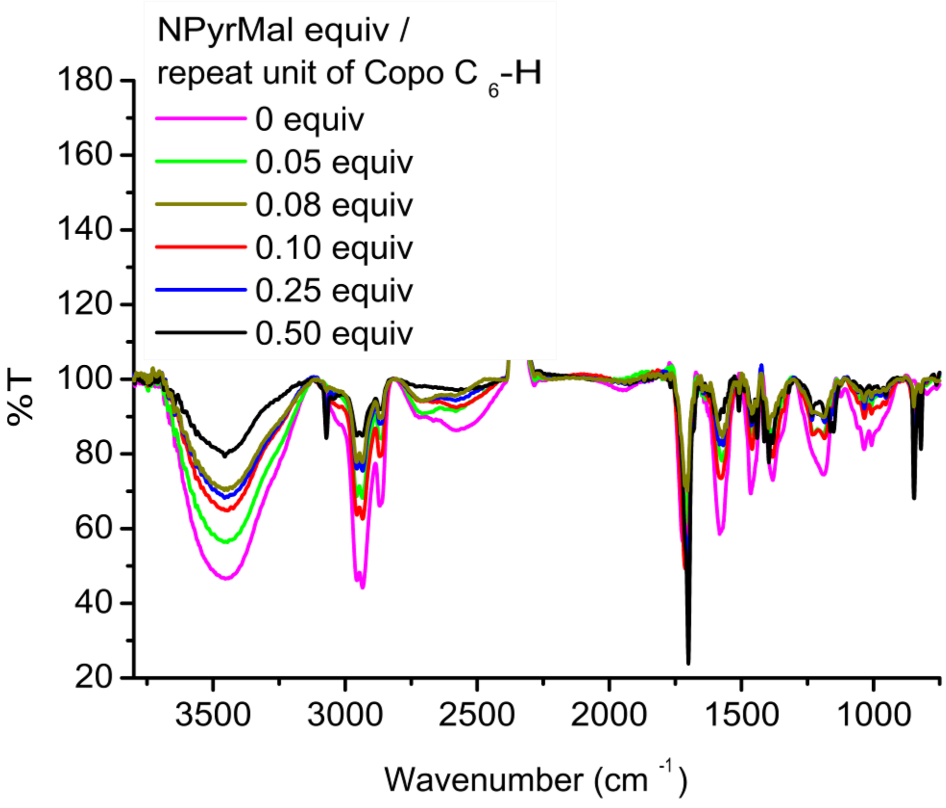

(**b**)

Table S2a. IR data for the $\nu_{C=O}$ band of carboxylic acid of the host for the Copo C_6-H/and Copo C_6-H/NPyrMal materials.

	**$\bar{\upsilon}$ (cm^{-1})**	**$\Delta\bar{\upsilon}$**

Copo C_6-H (host)		1717	/
Materials (NPyrMal equiv)	0.05	1714	-3
	0.08	1714	-3
	0.1	1712	-5
	0.25	1712	-5
	0.50	1712	-5

Table S2b. IR data for the $\nu_{C=O}$ band of imide group of the guest for the *N*-pyrenylmaleimide and Copo C_6-H/NPyrMal materials.

		**$\bar{\upsilon}$ (cm^{-1})**	**$\Delta\bar{\upsilon}$**
NPyrMal (guest)		1702	/
Materials (NPyrMal equiv)	0.05	/ [a]	/ [a]
	0.08	1705	+3
	0.1	1714	+2
	0.25	1702	0
	0.50	1701	+1 [b]

[a] νC=O (COOH, host) band superimposed with $\nu_{C=O}$ (imide, guest).[b] Not significant shift (Resolution = 0.8 cm^{-1}).

V. "Host-guest" materials PEG-5000/CCNPyr

V.1. X-Ray data

Figure S20. X-Ray difractogramms of "guest-host" materials PEG 5000/CCNPyr. For sake of clarity, each diffractogram with a positive CCNPyr loading was shifted of + 1500 cps one of the other.

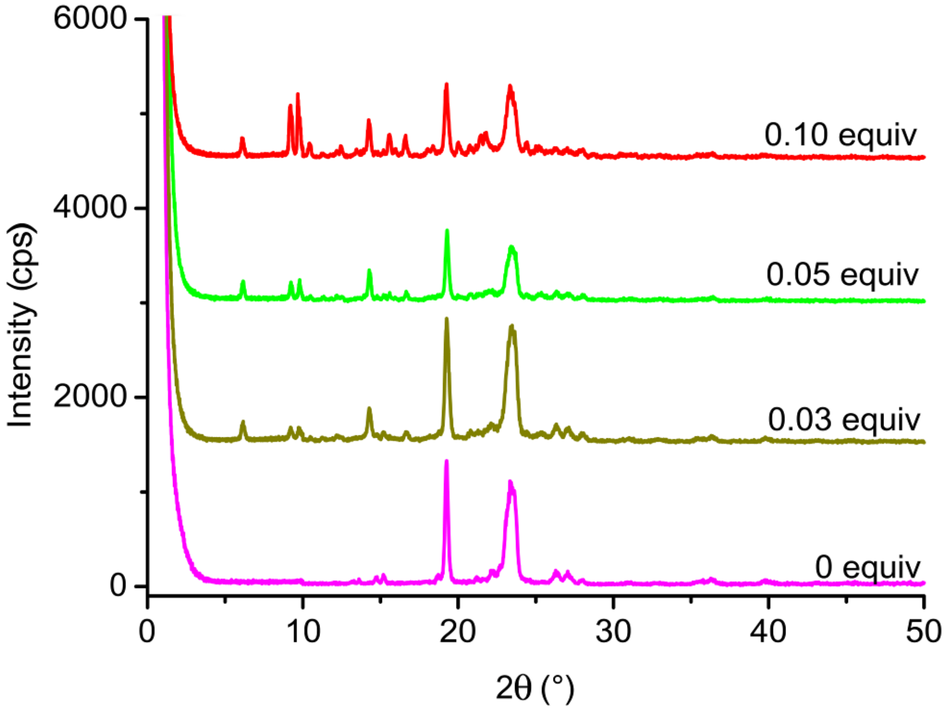

Figure S21. Schematic representation of the postulated non covalent interactions, in the solid state, (**A**) between pyrene and the Copo C_6-H matrix; (**B**) between *N*-pyrenylmaleimide and the Copo C_6-H matrix.

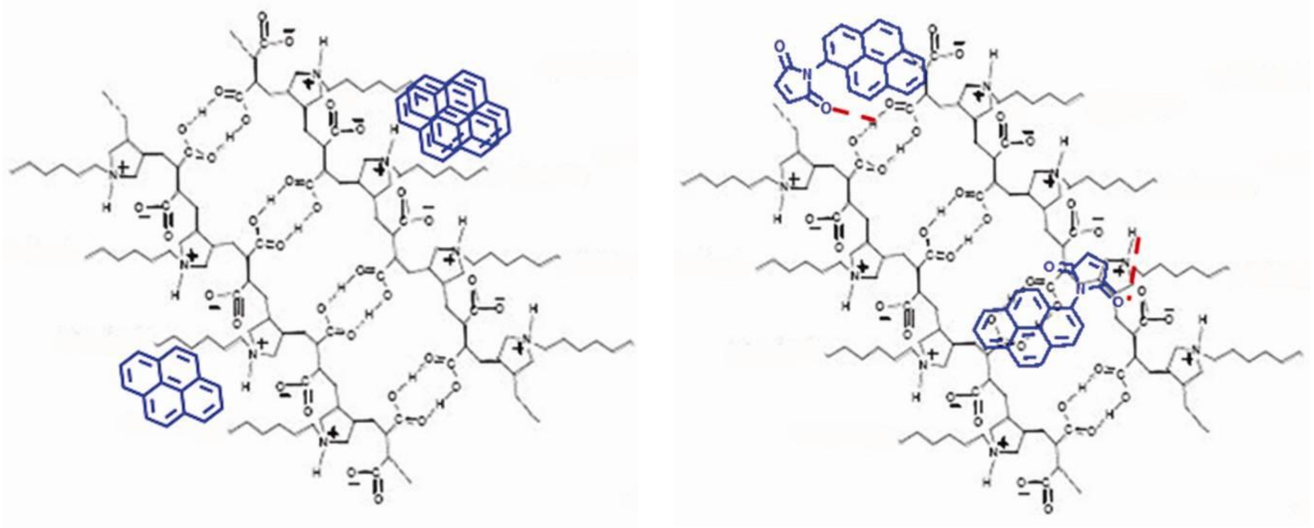

(**A**) (**B**)
